# Grasping Ability and Motion Synergies in Affordable Tendon-Driven Prosthetic Hands Controlled by Able-Bodied Subjects

**DOI:** 10.3389/fnbot.2020.00057

**Published:** 2020-08-26

**Authors:** Immaculada Llop-Harillo, Antonio Pérez-González, Javier Andrés-Esperanza

**Affiliations:** Grupo de Biomecánica y Ergonomía, Departamento de Ingeniería Mecánica y Construcción, Universitat Jaume I (UJI), Castelló de la Plana, Spain

**Keywords:** 3D printing, anthropomorphic hand, experimental analysis, functional testing, grasping, prosthetic hand, synergies

## Abstract

Affordable 3D-printed tendon-driven prosthetic hands are a rising trend because of their availability and easy customization. Nevertheless, comparative studies about the functionality of this kind of prostheses are lacking. The tradeoff between the number of actuators and the grasping ability of prosthetic hands is a relevant issue in their design. The analysis of synergies among fingers is a common method used to reduce dimensionality without any significant loss of dexterity. Therefore, the purpose of this study is to assess the functionality and motion synergies of different tendon-driven hands using an able-bodied adaptor. The use of this adaptor to control the hands by means of the fingers of healthy subjects makes it possible to take advantage of the human brain control while obtaining the synergies directly from the artificial hand. Four artificial hands (IMMA, Limbitless, Dextrus v2.0, InMoov) were confronted with the Anthropomorphic Hand Assessment Protocol, quantifying functionality and human-like grasping. Three subjects performed the tests by means of a specially designed able-bodied adaptor that allows each tendon to be controlled by a different human finger. The tendon motions were registered, and correlation and principal component analyses were used to obtain the motion synergies. The grasping ability of the analyzed hands ranged between 48 and 57% with respect to that of the human hand, with the IMMA hand obtaining the highest score. The effect of the subject on the grasping ability score was found to be non-significant. For all the hands, the highest tendon-pair synergies were obtained for pairs of long fingers and were greater for adjacent fingers. The principal component analysis showed that, for all the hands, two principal components explained close to or more than 80% of the variance. Several factors, such as the friction coefficient of the hand contact surfaces, limitations on the underactuation, and impairments for a correct thumb opposition need to be improved in this type of prostheses to increase their grasping stability. The principal components obtained in this study provide useful information for the design of transmission or control systems to underactuate these hands.

## Introduction

The advent of 3D-printing technology in the prosthetics or orthotics industries has led to the generation of affordable and customized designs. These designs attempt to meet the most basic needs in the shortest time and with the least amount of money [typically < $500 for a hand prosthesis (ten Kate et al., 2017)]. For the purpose of this research, being affordable refers primarily to prosthetic hands printed using fused deposition modeling (FDM) technology, which is becoming popular under the do-it-yourself (DIY) premise (RepRap, 2017). A large number of these hand designs can be freely downloaded from Computer-Aided Design (CAD) repositories such as www.instructables.com and www.thingiverse.com, or from government institutions such as the National Institutes of Health (NIH) 3D print exchange (U.S. Department of Health Human Services-National Institutes of Health, 2017). Non-profit initiatives such as Open Hand Project (Gibbard, 2013), e-NABLE (e-NABLE, 2014), or Openbionics (Gibbard, 2018) are the main sources for such repositories. Anyone can download a CAD model, typically a stereolithography file (.stl), and print it. Most designs require additional elements for final assembly, such as screws/bolts, elastic cords, nylon cords, and Velcro®, which should be readily available (Burn et al., 2016). Evidently, there is some debate about the fact that these designs may not be recognized as medical devices, since their manufacturers are not subject to any control by the National Regulatory Authorities (Asanuma, 2012a,b). Nevertheless, this undeniable trend of accessible production has motivated some reviews in recent years (Phillips et al., 2015; Burn et al., 2016; Tanaka and Lightdale-Miric, 2016; ten Kate et al., 2017) and continues to encourage deeper analysis. Owing to the lack of comparative studies on the functionality of this kind of prostheses, one of the aims of this paper is to address this gap in the literature.

Controzzi et al. (2014) specified six important issues to be considered during the design and development phases of a prosthetic hand. Having a deeper insight into the affordable designs but taking these issues into account can help to establish links between the design process and the usage environment:

- *Kinematic architecture*: refers to the mechanical concept in which degrees of freedom (DoFs) are related to the required degrees of actuation (DoAs), independently of issues (b) and (c). Underactuated mechanisms are those with fewer DoAs than DoFs, usually linking the movement among the joints of each finger.
- *Actuation principle*: all early affordable designs were body powered. While body-powered devices may perform multiple tasks, their fingers bend together and users have difficulty grasping the object as tightly as possible (Dally et al., 2015). DIY has also taken advantage of the latest open-source developments to accomplish electric prostheses, like the Arduino microcontrollers (www.arduino.cc), together with compact DC motors and compact batteries.
- *Actuation transmission* connects (a) with (b): the power transmission from the actuators to the fingers should be stiff during the flexion but avoid any damage due to haphazard impacts on the dorsum. The spontaneous adaptation to the shape of the grasped object is also desirable in terms of stability. These two reasons and the ease of assembly make the use of nylon threads running through sheaths the most common transmission in low-cost designs.
- *Sensors*: for the scope of affordable devices, both assembly and maintenance are far easier when the hand is used as an open-loop device.
- *Materials* and (f) *manufacturing method* are already strongly related to the FDM technology considered: acrylonitrile butadiene styrene (ABS) and polylactic acid (PLA) are the same thermoplastic materials as those used in conventional orthotics, with similar biocompatibility, stability, durability, and mechanical properties (Ventola, 2014; Burn et al., 2016). Moreover, the use of compliant materials such as Ninjaflex® in the manufacturing of joints may avoid the need to use an additional extension system for the fingers.

A compromise between the number of motors and the grasping ability of the prosthetic hands appears during the design process. Fewer motors allow reductions in weight and cost, but at the expense of less motion versatility. Although underactuation, from issue (a) may involve a loss of dexterity, it is the preferred architecture in a DIY context due to its ease of assembly and maintenance. As compensation and from the perspective of issue (b), the use of electric prostheses enhances the dexterity by controlling each finger independently. Moreover, the analogy of nylon threads acting like the tendons in the human hand, issue (c) also allows the actuators to be located remotely, in the palm or forearm space, thereby reducing the dimensions and weight of the fingers.

In this study, the hands selected meet the common characteristics described above, that is, with no feedback from sensors, tendon transmissions as the preferred option, and being made of regular FDM materials. How their designers resolved the issues outlined previously will be described below, together with our own experience in the assembly process.

The analysis of the motion synergies or *eigenpostures* among fingers for a hand with more DoFs than DoAs could be a method to aid in decision-making in the early stages of the design. In the literature (Santello et al., 2016), synergies have been successfully applied to create novel design and control concepts for artificial hands. Roboticists have proposed synergies applicable to the pre-grasping phase in order to reduce the number of DoFs that can be controlled in an independent manner. The fingers of the affordable hands for which the motions of the tendons are highly correlated can be candidates to be moved by a single controller, or even a single actuator. The recent reviews by Salvietti (2018) and Santello et al. (2016) prove that the approach of replicating human hand synergies in robotic hands has been the aim of some studies in the last decade. The review by Salvietti (2018) shows the main solutions in the literature for reducing dimensionality by coupling some DoFs of the robotic hands, at both software and hardware levels. Most of them were based on the human hand postural synergies defined in Santello et al. (1998). According to the software synergies, two main categories were highlighted by Salvietti: (i) mapping of synergies from humans to robots using the data collected from the human hand and (ii) defining the synergies for robotic hands by collecting data from grasps obtained directly with the robotic hand. From this study, the first category may make it possible to benefit from the highly evolved control model that the human brain is, but it has the difficulty of adapting the data to the kinematics of a robotic hand. The second category allows very specialized synergies to be obtained for the specific hand kinematics but may depend heavily on the set of grasps selected. However, to our knowledge, no previous work, with the exception of a preliminary study by the authors (Llop-Harillo and Pérez-González, 2017b) on a hand prototype, has analyzed the motion synergies of tendon-driven prosthetic hands (TDPHs) with each DoA being controlled by a finger of a healthy human subject, that is, taking advantage of the human brain control mechanisms while obtaining the data directly from the artificial hand. Such data would summarize the positive characteristics remarked by Salvietti (2018) and would therefore be specialized for the specific device kinematics. This approach is used in the present study, and a comparison of the synergies obtained for different TDPHs will be analyzed in the paper.

In the research literature, most upper limb prostheses are still evaluated through subjective protocols or questionnaires on end-users (Lindner et al., 2010). However, the use of an able-bodied adaptor (ABA) to adapt the prosthesis to a healthy subject has proven its usefulness in preliminary functional assessments (Kyberd, 2011; Dalley et al., 2012; Bouwsema et al., 2014; Fougner et al., 2014; Vasluian et al., 2014; Smit et al., 2015; Huinink et al., 2016; Rossi et al., 2017) prior to any testing involving more sensitive potential users. The majority of these functional assessments used objective protocols such as the Southampton Hand Assessment Procedure (SHAP) (Light et al., 2002), the Box and Block Test (BBT) (Mathiowetz et al., 1985a), or the Nine-Hole Peg Test (NHPT) (Mathiowetz et al., 1985b). Notwithstanding, all these tests are designed and commonly used to assess the human hand function. Moreover, BBT and NHPT use a limited variety of grasping types and objects. In a recent study by the authors (Llop-Harillo et al., 2019), a benchmark to assess the grasping ability of anthropomorphic artificial hands was presented. This benchmark is composed of 26 tasks involving grasping with the eight most relevant human grasp types (GTs) during activities of daily living (ADLs) and two non-grasping postures. The set of grasps selected in this benchmark account for more than 90% in grasp frequency according to several previous studies found in the literature (Sollerman and Ejeskär, 1995; Light et al., 2002; Bullock et al., 2013; Vergara et al., 2014; Feix et al., 2016; Wang et al., 2018).

In this study, we address some questions resulting from the approaches presented above: Are the publicly available 3D-printed prostheses functional to perform ADLs? Are there any significant differences in functionality among existing models or the intended GT? What are the main limitations from a mechanical point of view for functional grasping with these prostheses? Is the effect of the subject on the grasping ability of the hands significant when using an ABA? What are the motion synergies on TDPHs with a human control strategy?

The objective of this paper is to evaluate and compare the functionality and human-like grasping of several affordable 3D-printed TDPHs using a publicly available experimental protocol published by the authors (Llop-Harillo et al., 2019). The main difficulties these artificial hands have to perform the most common grasps in ADLs are analyzed. A custom-made ABA is used to control each of the hand tendons independently with the motion of a healthy subject's fingers. In this way, the control of the hand is performed by the subject, and the specific solutions adopted by the designers for controlling or underactuating the hand are excluded from the comparison. Thus, the hands are compared in terms of their kinematic chain, finger segments geometry, and the characteristics of the contact surfaces: materials, stiffness, and friction coefficients. Additionally, the ABA includes suitable electronics to measure and register the displacement of the tendons during the grasping process, thereby enabling the analysis of the motion synergies employed. Another objective of this paper is to analyze the effect of the subject on the control of these TDPHs using the ABA.

## Materials and Methods

### Tendon-Driven Prosthetic Hands

Three of the most common 3D-printed electrical prosthetic hands for transradial amputees currently available online, with documented clinical usage, were chosen for the present study, namely (Andrés et al., 2019), the Dextrus v2.0, the InMoov, and the Limbitless hands. Together with the IMMA hand, designed by the authors (Llop-Harillo and Pérez-González, 2017b), Table 1 summarizes each of the mechanical design characteristics as outlined by Controzzi et al. (2014) and summarized in the previous section. Note that the *actuation transmission* was performed with nylon threads for all of them, none of them have any *sensors*, and the *manufacturing method* was FDM. As they try to mimic the anatomy of the human hand, the joints of the fingers and thumb are named by analogy, from distal to proximal: distal interphalangeal (DIP), proximal interphalangeal (PIP), metacarpophalangeal (MCP), and carpometacarpal (CMC). Details regarding the actuation principles are those obtained from the download source together with the CAD model. All hands were printed using a Colido® mod. X3045 printer with Repetier-Host software. Prior to that, a wrist supplement was added to each hand in order to make it easier to fasten the device to the ABA. Time and cost have been estimated on the basis of the printer model and materials employed. Note that the same 0.8-mm nylon fishing line (ultimate tensile strength of 220.5 N) was employed as a tendon for each finger of all the hands. It runs through all the phalanges up to the distal end of the finger, where it is knotted. Further details are provided in Table 1 and in the following paragraphs.

**Table 1:** Main design characteristics of the selected 3D-printed hand prostheses.

Hand model (download link)	IMMA (Llop-Harillo and Pérez-González, 2017a) 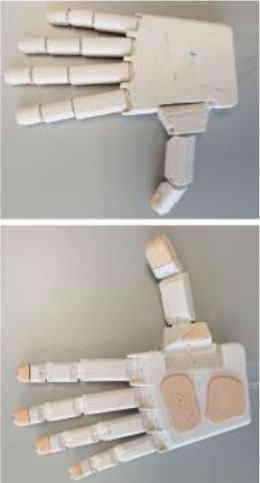	Limbitless (UCFArmory Enablingthefuture, 2014) 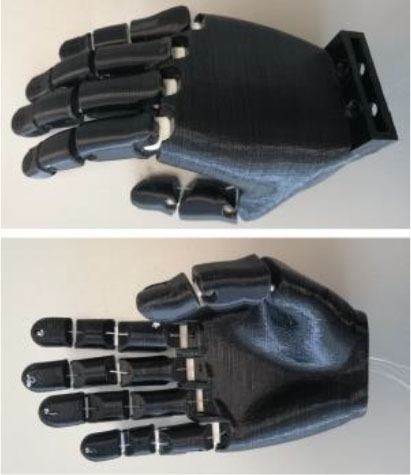	Dextrus v2.0 (Gibbard, 2015) 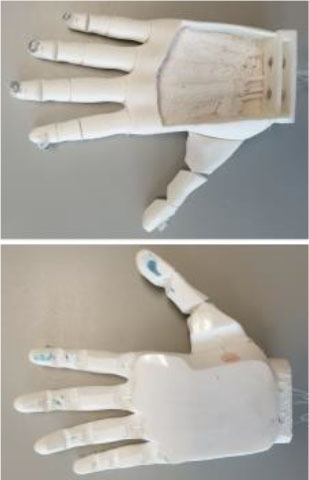	InMoov (Langevin, 2013) 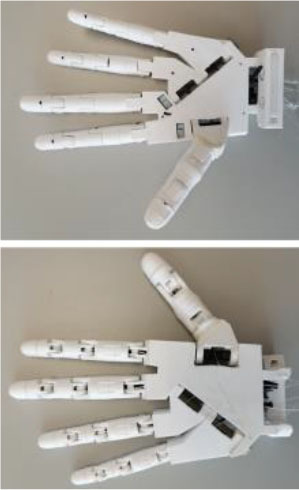
Kinematic architecture^*^	Underactuated (15 DoF > 6 DoA)	Underactuated (14 DoF > 1 DoA)	Underactuated (15 DoF > 5 DoA)	Underactuated (17 DoF > 5 DoA)
Number of joints	3f [DIP, PIP, MCP] 3th [DIP, MCP, CMC]	3f [DIP, PIP, MCP] 2th [DIP, MCP]	3f [DIP, PIP, MCP] 3th [DIP, MCP, CMC]	3f [DIP, PIP, MCP] + 1 [CMC at ring and little fingers] 3th [DIP, MCP, CMC]
Actuation principle	Not defined	1 Servo motor (Hitec HS-5645MG—Digital high torque MG servo)	5 DC linear actuators (Actuonix PQ12-63:1 linear actuator)	5 Servo motors (either HobbyKing HK15298, Tower Pro MG995, or equivalent)
Motor location^**^	–	Farm	Palm	FArm
Materials (infill)	PLA-Soft® (20–40%)/Filaflex® (pads) (20–30%)/NinjaFlex® (joints) (40%)	PLA (25%)/Ninjaflex® (joints) (25%)	Ninjaflex® (35%)	PLA (30%)
Total weight (g)	131.5	144.5	131	201.5
Cost 3D-printing materials	$9	$6	$11	$6
Printing time	45 h	16 h	28 h	22 h
Dimensions^***^ (HL/HB) (mm)	184/80	200/89	185/87	194/95
Clinical usage	–	Limbitless Solutions, 2015; Owen, 2018	Alec, 2015	Huchet, 2014

* *3f, three joints at fingers; 3th, three joints at the thumb; 2th, two joints at the thumb. DoA as originally intended for each model*.

** *Palm, inside palm (or on palm dorsum for control board and battery); FArm, Actuators/control board/battery in forearm*.

*** *HL, hand length (from the most proximal palmar point to the tip of the middle finger); HB, hand width (at the metacarpal heads)*.

#### IMMA Hand

The IMMA hand is a tendon-driven anthropomorphic prosthetic hand prototype with six DoAs, designed at the Universitat Jaume I (UJI) by the authors (Llop-Harillo and Pérez-González, 2017b). Flexion and circumduction movements of the thumb are actuated separately with two different nylon threads, allowing opposition of the thumb to orient its distal phalanx toward the distal phalanges of the fingers. Therefore, it contains a total of six tendons: five for the flexion of the fingers and an additional one for the circumduction of the thumb. For the sake of simplicity in the assembly, and similarly to the Limbitless Hand, it uses elastic elements at the joints to drive extension movements when the tendons are released. Its main dimensions are between the 50th percentiles of the male and female human hands, based on data obtained in the authors' research group (Vergara et al., 2018).

It was 3D-printed by FDM combining PLA SOFT-Flexible® [a mixture of PLA and thermoplastic polyurethane (TPU) using hexamethylene diisocyanate] for the palm and phalanges, NinjaFlex® (special formulation of TPU with high flexibility and durability) for the elastic joints, and FilaFlex® (based on TPU with additives) for the finger and palm pads. The pads located in the main areas of contact with objects are intended to mimic the friction coefficient of the skin of the human hand. The IMMA hand is licensed under a Creative Commons (CC BY-NC-ND 4.0: Attribution-Non-Commercial-NoDerivatives) license.

#### Hand of the Limbitless Arm

Developed by the University of Central Florida Armory, the Limbitless Arm (Limbitless Solutions, 2015) is the first myoelectric design available from the e-NABLE site (e-NABLE, 2014). It is based on the body-powered Flexy-Hand (Steve, 2014). It is available in two versions in the same download (UCFArmory Enablingthefuture, 2014): with no palmar abduction of the thumb and with the thumb with a palmar abduction of 45°. This latter was the model used in the present research. It has five tendons, one for the flexion of each finger, and it is originally intended to work with one actuator, thus closing fingers and thumb together. For the purposes of this research, however, this issue is overlooked, and each finger is operated independently, as in the other models. The Limbitless arm is licensed under a Creative Commons (CC BY-NC 3.0: Attribution-Non-Commercial) license.

#### Dextrus v2.0

The second version of the Dextrus hand, available at the Open Hand Project website (Gibbard, 2015), showed significant changes with regard to the first version. It is made entirely of flexible material (Ninjaflex®), and so it has flexible joints fully integrated within the design. It has five tendons, one for the flexion of each finger, and uses five linear actuators embedded in the palm. This initial advantage could make it difficult to resize for smaller hands, for example for children. Nevertheless, it was printed in its default size. For this research, the linear actuators are not included, and the tendons are extended to the forearm for actuation with the ABA described below.

Its rubberized unibody design makes this hand very easy to assemble: the tendons only need to be routed to make the fingers mechanically compliant when being forced to close. It makes replacing individual fingers impossible if broken. Dextrus v2.0 is licensed under a Creative Commons (CC BY-SA 4.0: Attribution-ShareAlike) license.

#### InMoov

The InMoov prosthetic hand (Langevin, 2013) was originally launched in 2012, as a part of an Open Source 3D printed life-size robot. Each finger can be mounted in such a way as to achieve an active two-way control: flexion and extension. For the sake of simplicity and to evaluate grasping capabilities, an elastic band was used here for finger extension. This hand has five tendons for the flexion of the fingers but with the particularity of additional joints in the little and ring fingers close to the center of the palm in order to reproduce the palmar arch. Cut bike spokes were used for thumb and finger joints and a bolt for the palmar joint. The InMoov hand is licensed under a CC BY-NC 3.0 license.

### Experiments

For this research, each affordable 3D-printed prosthesis was confronted with the Anthropomorphic Hand Assessment Protocol (AHAP) (Llop-Harillo et al., 2019), described briefly below. Three able-bodied subjects assessed the performance of the four hands using the ABA, described in a subsequent section. All subjects were right-handed, and they adapted the prostheses to their right arm. All of them had a similar user experience with the ABA and the artificial hands. Once the electrical actuation of the hands had been removed, the ABA allows the user to move each DoA of the artificial hand by using their fingers to pull the individual cords (tendons) with a ring attached to the end of each of them. This actuation method allows any control and actuation issue to be kept separate from the evaluation of the mechanical performance of the artificial hand and transfers the control of the artificial hand to the human brain.

Each hand was fastened to the ABA, and then, the device was tested by the same three subjects before fastening and testing a different hand. The order in which the hands were tested was IMMA, Limbitless, Dextrus v2.0, and InMoov. It should be noted that, in this within-subject design of the experiment, learning and transfer across trials is to be avoided. To do so, the subjects chosen were already familiarized with the use of the ABA itself as they participated in its design. This familiarization is not a difficult process, as it involves a natural task of pulling the corresponding thread for each finger, with visual, haptic, and proprioceptive feedback. This situates the subject's learning curve on a plateau. Moreover, to reinforce this plateau effect, in each session and after attaching the prosthesis to the subject's forearm using the ABA, the subject was given some time to return to the ABA actuation and then carried out the AHAP following the experimenter's instructions. Finally, to avoid fatigue effects and to minimize learning effects, each subject tested each prosthesis in a different session, with a time gap of at least 40 days between them.

Each of the 12 experiments (3 subjects and 4 prostheses) was video-recorded and lasted ~60–110 min. The Ethics Committee of the UJI approved the study, and written informed consent was obtained from all the participants. The three adult male subjects who participated in the experiment were free from hand pathologies or injuries (see characteristics in Table 2; the grip strength of their right hand was measured with a CAMRY® Digital Hand Dynamometer).

**Table 2:** Characteristics of the study sample.

**Subject**	**Age**	**Hand length (mm)**	**Hand width (mm)**	**Grip strength (kgf)**
S1	53	190	91	44.2
S2	24	194	94	39.3
S3	39	181	86	48.7

#### Anthropomorphic Hand Assessment Protocol

**Figure 1:**
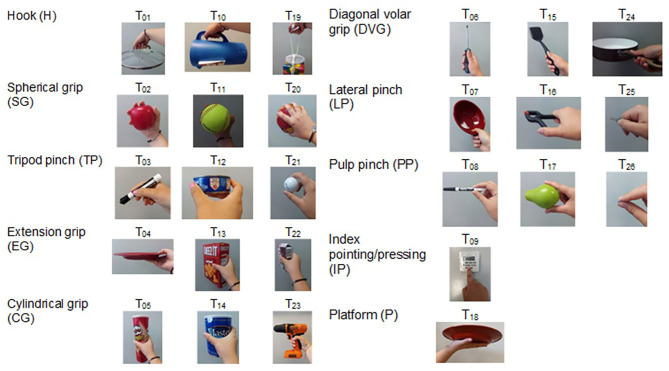
Tasks (T_i_; i: indicates the task order in the protocol) of the Anthropomorphic Hand Assessment Protocol (AHAP) (Llop-Harillo et al., 2019).

The AHAP (Llop-Harillo et al., 2019) is a validated benchmark to quantify the grasping ability of anthropomorphic hands. It is composed of 26 tasks (Figure 1) involving the eight most common human GTs in ADLs: pulp pinch (PP), lateral pinch (LP), diagonal volar grip (DVG), cylindrical grip (CG), extension grip (EG), tripod pinch (TP), spherical grip (SG), and hook (H); and two non-grasping postures: platform (P) and index pointing/pressing (IP). Three objects were used per GT and one for each non-grasping posture. These objects were selected with different sizes, shapes, weight, texture, and rigidity from the publicly available Yale–CMU–Berkeley Object and Model Set (Calli et al., 2015). For each task, the object is handed over to the subject by an experimenter holding it in the correct position for the successful execution of the grasp. The experimenter then releases it once the grasp has been performed with the artificial hand. Prior to the trials to be evaluated, the experimenter indicates the correct grasping posture for each object/task and the subject should try to reproduce it as accurately as possible after 1 min of pre-practice. In the test, the subject has to first grasp the object released by the experimenter with the palm facing upwards and then hold the object for 3 s. If the grasp is completed with the correct GT, the score for that trial is 1 point; if the grasping posture is different from the one specified, the score is 0.5 points; and if the artificial hand cannot hold the object, the score is 0. In the first two cases and with the object still grasped, the next step of the protocol consists in rotating the wrist of the subject 180° for the palm facing downwards and holding the object for three additional seconds in this pronated position. For this step, if there is no visible motion of the object with respect to the hand, the score is 1 point; if the object moves with respect to the hand but is not dropped, the score is 0.5; and if the object is dropped, 0 points. These steps must be repeated three times for each object. This benchmark allows quantification of the functionality and human-like grasping of artificial hands through a numerical Grasping Ability Score (GAS). Its reliability, consistency, and responsiveness have been statistically validated (Llop-Harillo et al., 2019). To obtain the GAS for each hand, the AHAP considers both grasp correctness (human-like) and stability after rotating the artificial hand from supination to pronation. For a better analysis, the GAS score can be split into two terms corresponding to the grasp correctness (*grasping*) and the stability under motion (*maintaining*). As the GAS is defined as a normalized score, it can be expressed as a percentage of human grasping ability, with 100% corresponding to the healthy human hand. Moreover, for each of the 10 GTs/postures in the AHAP, the scores for the three related objects can be added to obtain a “partial GAS” for each GT. These partial GAS are also normalized and expressed as a percentage of the maximum possible score, as for the GAS (see Llop-Harillo et al., 2019).

#### Able-Bodied Adaptor

**Figure 2:**
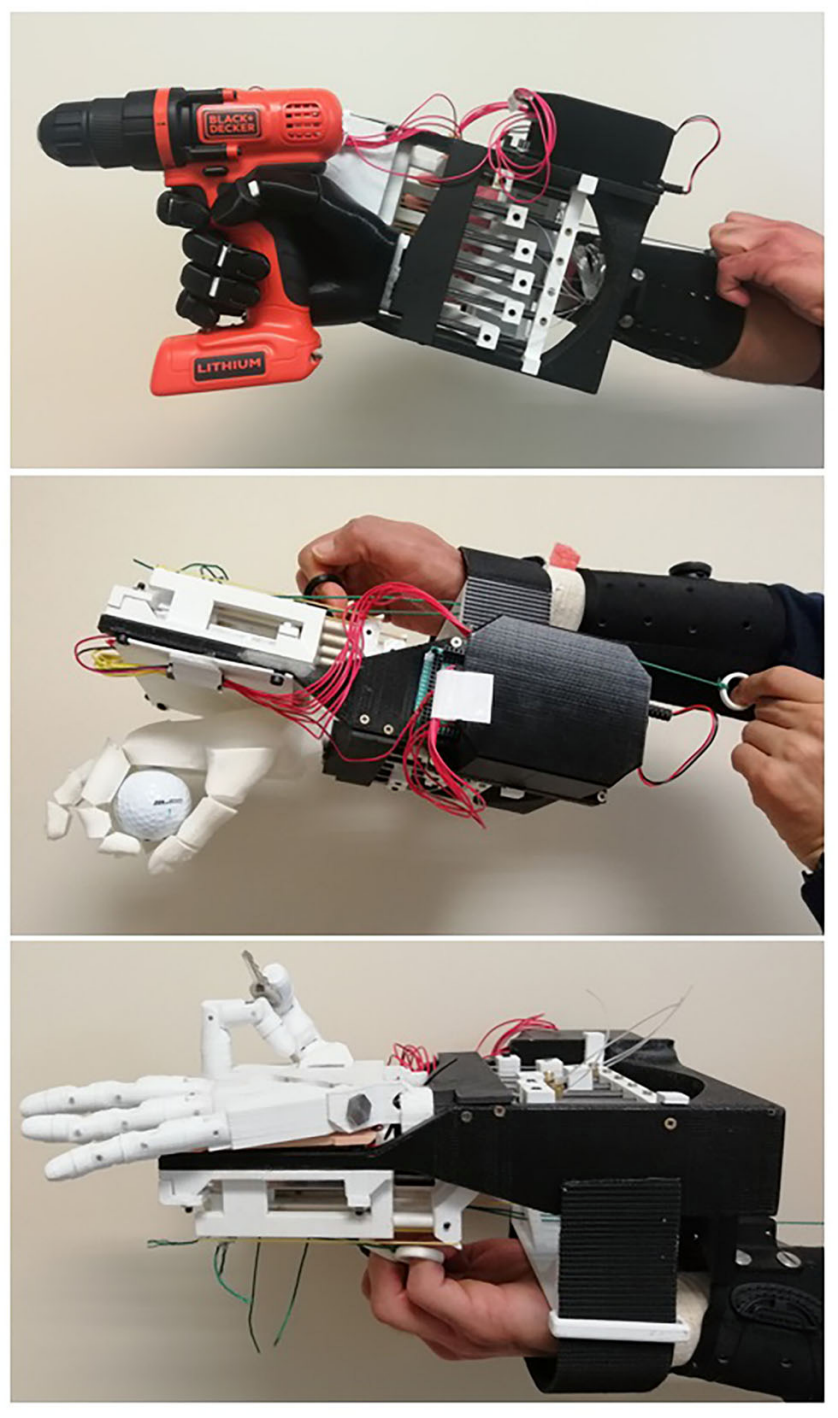
Different views of the able-bodied adaptor (ABA).

A specifically designed adaptor was needed to perform the AHAP with TDPHs controlled by healthy human subjects. In a previous initial study by the authors (Llop-Harillo and Pérez-González, 2017b), a preliminary design of an ABA was developed to measure the cable excursion of TDPHs when they were controlled by a human subject. However, one of the problems of this earlier ABA was that the final position of the artificial hand was about 35 cm more distal than the user's sound hand, thus forcing them to perform unnatural compensatory movements of the arm. For the present study, that ABA was redesigned, leading to a lower distal separation of the artificial hand with respect to the user's own hand (Figure 2). In addition, the friction introduced in the tendon motions in the current design is lower than in the previous version. It consists of a TRS prosthetics' Pro Cuff® and a PLA 3D-printed structure of our own design. The ABA is to be connected to the forearm of an able-bodied subject and allows them use their fingers to control any TDPH moved by up to six tendons. It registers the tendon excursions during the motion of the hand through six BOURNS® PTB6043-2010BPB103 linear potentiometers, with a measurement range of 60 mm, connected to an Arduino® Mega 2560 board. The system also includes an HC-05 Bluetooth module, which enables wireless communication. The electronics are powered by a 9-V battery, which is connected to the power jack of the Arduino board.

#### Acquisition of Grasping Data

The data acquisition software developed permits registration of the displacement of the six linear potentiometers within the ABA, thereby measuring the displacement of the different tendons that actuate the artificial hand during the tests performed. For this purpose, the Arduino board reads the values of the six potentiometers using six analog inputs and sends these values to a laptop computer by Bluetooth® communication. Using Matlab®, these measures are stored in a matrix along with the time. The data recording process is divided into tasks and repetitions.

### Data Analysis

First, the GAS obtained with the different artificial hands was analyzed and compared. For a deeper interpretation of the results, the GAS was also analyzed, splitting the score into the two aspects corresponding to *grasping* and *maintaining* steps of the protocol (see the section above describing the AHAP). Furthermore, the partial GAS corresponding to each GT/posture was also compared for the different hand prostheses.

Second, as we used an ABA to fit the artificial hands to the subject's arm and to actuate them, the experiment includes two different factors that could affect the GAS obtained from the test: (i) the hand design and (ii) the subject's ability to control the artificial hand using the ABA. All the hands were tested by all the subjects, corresponding to a full factorial design. Thus, a full factorial two-way analysis of variance (ANOVA) on the GAS with factors “subject” (3 levels) and “hand” (4 levels), and a full factorial three-way ANOVA on “partial GAS” with factors “subject” (3 levels), “hand” (4 levels), and “GT/posture” (10 levels) were conducted to ascertain the effect of the different factors involved in GAS and partial GAS, respectively. It should be noted that the subject effect was included as an independent factor in the analyses in order to quantify the variance associated with this factor and to be able to evaluate its significance in the results. A *post hoc* analysis [honestly significant difference (HSD) Tukey] was performed to gain a deeper insight into the significant differences between the levels of those factors found to be statistically significant in the ANOVA tests.

Third, a group of analyses using the Pearson correlation coefficient (CC) and principal component analysis (PCA) among tendon displacements was undertaken to study the motion synergies among fingers in the different hands. For the analysis of the correlation between tendon displacements in a grasping task repetition, the instantaneous displacements recorded from the start of the task to the achievement of the grasp were stored in a data matrix with columns corresponding to the different potentiometers (tendons). To avoid unwanted effects derived from noise in the potentiometer signals, displacements of <0.2 mm were rounded to 0. For each trial, the grasp was considered to have been achieved once all the tendons reached their maximal displacement, excluding inactive tendons, during the grasp. For the subsequent analyses, only the data of the grasps performed successfully (those with a score of 1 or 0.5 in the *grasping* step of the AHAP) were considered. For each successful repetition of each task, the CC between any pair of columns of the data matrix was computed. The correlation between a pair of tendons was considered high in a grasping task repetition if the CC of the corresponding columns was above 0.9. For each pair of tendons, the percentage of successful grasps for which the CC was >0.9 was a measure of the tendon-pair synergy (TPS). A TPS close to 1 indicates that this pair of tendons could be controlled with the same actuator, while a TPS close to 0 indicates a null correlation for this tendon pair. TPSs were compared for the different hands and subjects. A two-way ANOVA on TPS with factors “subject” and “tendon pair” was conducted for each hand.

The data matrices of tendon displacements obtained with the procedure described above and corresponding to all tasks and repetitions with successful grasping were stacked in a single matrix for each hand and subject. A PCA was then conducted on these stacked data matrices of tendon displacements. The PCA could make it possible to reduce the dimensionality corresponding to the tendon displacements in order to design underactuation systems for simpler control of the hands. The PCA was performed in two different ways: separately for each subject and including all the data for the three subjects together.

Matlab® was used to obtain the data matrices to compute TPSs, and the CC was computed with the built-in function *corrcoef*. The SPSS statistical package (version 25, SPSS Inc, Chicago, USA) was used for the statistical analyses. The PCA extraction method was based on a fixed number of factors to explain more than 80% of the variance, and the rotated component matrices were obtained using Varimax with Kaiser normalization as the rotation method.

## Results

### Grasping Ability Score

Table 3 shows the mean GAS and its standard deviation across subjects, obtained with the AHAP for each prosthetic hand analyzed. Moreover, it includes the detail of the scores corresponding to the observation of the grasp correctness and stability under motion, *grasping* and *maintaining*, respectively, as explained in Anthropomorphic Hand Assessment Protocol. All the results in Table 3 are shown as a percentage of their respective maximum possible score, where the maximum score (100%) for *grasping* is 78 points (26 tasks by 3 trials), 75 points for *maintaining* (25 tasks by 3 trials; the platform task is not scored in this step of the protocol), and a total of 153 points for the GAS.

**Table 3:** Grasping ability score (GAS ± SD) obtained with the AHAP where all the results are shown as a percentage of their respective maximum possible score.

**Hand**	**GAS (%)**	**Grasping (%)**	**Maintaining (%)**
IMMA	57 ± 2	77 ± 1	37 ± 4
Limbitless	50 ± 3	63 ± 2	37 ± 4
Dextrus	48 ± 4	61 ± 3	34 ± 6
InMoov	49 ± 1	57 ± 1	40 ± 2

These results show that the grasping ability of each of the four hands was below 60% with respect to the human hand (100%). It is relevant that in the *grasping* evaluation of the AHAP, the scores were over 55% in all hands, whereas in the *maintaining* evaluation, they were below 40%. This result reveals that holding the object securely under motion of the hand is more demanding than just adopting a grasping posture in a human-like manner. IMMA was the hand that scored the highest GAS (57%), largely because of its high *grasping* ability (77%). Dextrus v2.0 scored the lowest GAS (48%), in accordance with its low abilities to hold objects (34% for *maintaining*).

**Figure 3:**
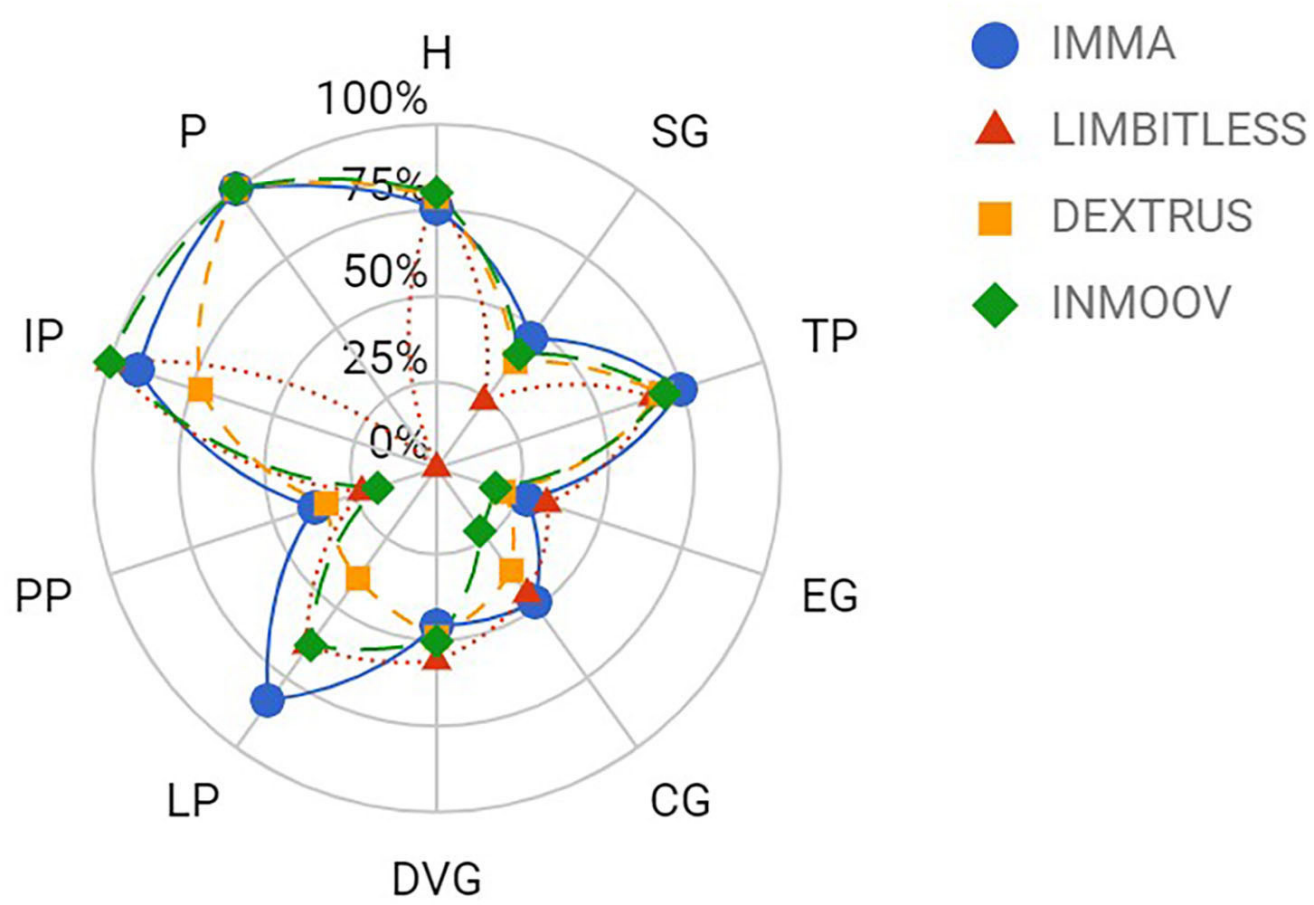
Mean partial GAS grouped by GT (PP, pulp pinch; LP, lateral pinch; DVG, diagonal volar grip; CG, cylindrical grip; EG, extension grip; TP, tripod pinch; SG, spherical grip; H, hook; P, platform; IP, index pointing/pressing) obtained by each prosthesis through the AHAP.

Figure 3 shows the partial GAS of the analyzed TDPHs for each common GT/posture in ADLs. It can be noticed that the platform posture was carried out perfectly (100%) by all hands except the Limbitless, due to the orientation and position of its thumb. The index pointing/pressing posture, needed for example to type with a keyboard, is the one that obtained the best results on average for the four hands analyzed, followed by hook and tripod pinch, with scores over 70%. However, the partial GAS for extension grip and pulp pinch was below 30%. The low results for extension grip can be explained by the difficulty, according to the mechanical design of TDPHs, in keeping the distal segments of the fingers extended while flexing their MCP joints. In the case of the pulp pinch, the issues arise due to the limitations when it comes to achieving the correct opposition between the thumb and the index finger, which relies on the orientation of the rotation axes of the thumb. The differences shown among the TDPHs analyzed in the lateral pinch are also attributed to the design of the thumb opposition but, in this case, regarding the radial side instead of the palmar side of the index finger. The additional DoA of the thumb for the IMMA hand is reflected in a better performance for pulp pinch, tripod pinch, and especially for lateral pinch. This problem of the orientation and mobility limitations of the thumb, together with the limitations imposed on the palm opposition by almost flat palm designs, also led to a discrete performance in the diagonal volar grip (around 50% in partial GAS). The limited grasping force that can be exerted is an additional problem in power grasps such as diagonal volar grip, extension grip, spherical grip, and cylindrical grip, especially with bigger or heavier objects. Moreover, for grasp stability in the different GTs, the contact surface on the fingers plays a very significant role. The low compliance and low friction coefficient that were observed in the hand–object contact areas for the TDPHs analyzed also affected the results.

The ANOVA tests (Tables 4, 5) showed that the effect of the “subject” factor was non-significant on both the GAS (*p* = 0.234) and on the partial GAS (*p* = 0.786). By contrast, the effect of the “hand” factor was found to be significant on both the GAS (*p* = 0.010) and the partial GAS (*p* = 0.037). The sum of squares and *F* statistics of the “hand” factor was very high compared to that of the “subject” factor. The *post hoc* analysis showed that the IMMA hand performed significantly better than the other three hands and that the differences among the other three hands were non-significant. The estimated marginal means and confidence intervals for the “hand” factor are shown in Table 6. Moreover, the results for partial GAS were significantly different (*p* < 0.001) depending on the GT/posture. An analysis by GT/posture showed that the partial GAS changed significantly with the hand for cylindrical grip (*p* = 0.002), diagonal volar grip (*p* = 0.027), and lateral pinch (*p* = 0.009). It is relevant to note that, for these three GTs, the partial GAS had intermediate values, namely, mean values of 38% for cylindrical grip, 50% for diagonal volar grip, and 63% for lateral pinch. The partial GAS for the platform posture was also significantly different for the Limbitless (0%) as compared to the other hands (100%). For the cylindrical grip, the InMoov obtained a significantly worse result; for the diagonal volar grip, the Limbitless obtained a better result than the other hands, and the IMMA achieved the worst; for the lateral pinch, the IMMA obtained a better result, and the Dextrus v2.0 achieved the worst. In none of the GT/postures did the subject have a significant effect on the partial GAS.

**Table 4:** Analysis of variance on GAS.

**Source**	**Type III sum of squares**	**Df**	**Mean square**	***F***	**Sig.**
Corrected model	194.670^a^	5	38.934	6.625	0.020
Subject	21.947	2	10.974	1.867	0.234
Hand	172.722	3	57.574	9.797	0.010
Error	35.259	6	5.877		
Total	31452.130	12			
Corrected total	229.929	11			

a *R squared = 0.835 (adjusted R squared = 0.697)*.

**Table 5:** Analysis of variance on partial GAS.

**Source**	**Type III sum of squares**	**Df**	**Mean square**	***F***	**Sig.**
Corrected model	60347.385^a^	14	4310.527	12.529	0.000
Subject	166.077	2	83.039	0.241	0.786
Hand	3034.480	3	1011.493	2.940	0.037
Grasp type	57146.828	9	6349.648	18.456	0.000
Error	36125.030	105	344.048		
Total	468389.211	120			
Corrected total	96472.414	119			

a *R squared = 0.626 (adjusted R squared = 0.576)*.

**Table 6:** Estimated marginal means and confidence intervals of GAS for factor “hand.”

		**95% Confidence interval**	
**Hand**	**Mean**	**Lower bound**	**Upper bound**
IMMA	57.407	53.982	60.831
Limbitless	50.217	46.792	53.641
Dextrus	47.823	44.399	51.248
InMoov	48.587	45.162	52.011

### Motion Synergies

The results of the ANOVA tests on TPS showed that the subject effect was non-significant (*p* = 0.280) for the IMMA hand, but it was significant for the other hands, with *p* = 0.017 for the Limbitless, *p* = 0.049 for the Dextrus v2.0, and *p* = 0.019 for the InMoov models. Moreover, for all the hands, the tendon pair was a significant factor in TPS.

**Figure 4:**
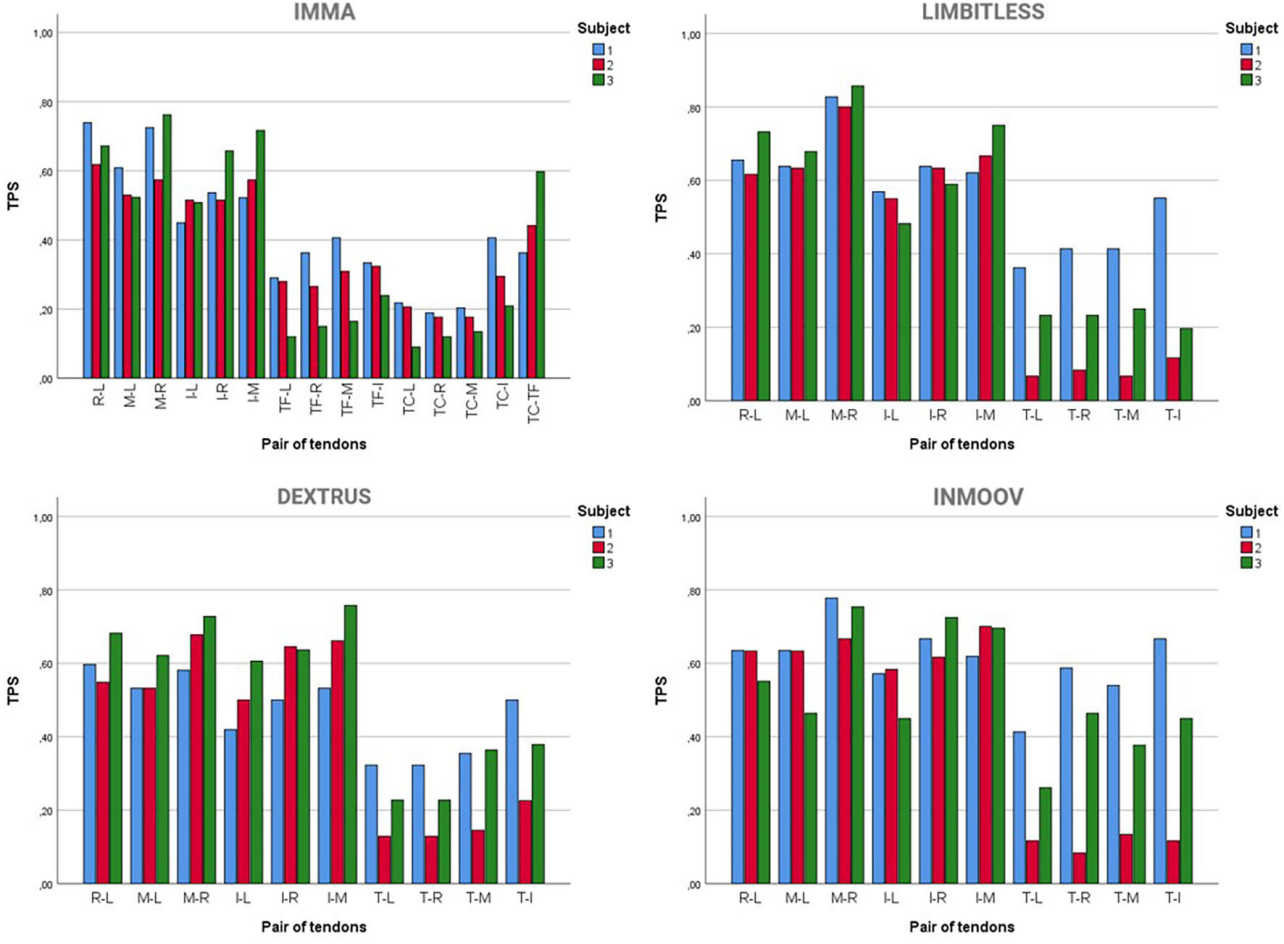
Tendon-pair synergies (TPSs) for each pair of tendons (L, little; R, ring; M, middle; I, index; T, thumb; TF, thumb flexion; TC, thumb circumduction) during successful grasping tasks in the AHAP for the four hands tested.

Figure 4 shows the TPSs for each tendon pair, per subject, for all the hands analyzed. For all the hands and subjects, the highest TPSs were obtained for pairs of long fingers (around 50–70% on average), showing that the subjects moved the tendons for the long fingers in a coordinated manner (CC > 0.9) for an important fraction of the successful grasps. The coordination between index and little fingers is always lower than that between the rest of the pairs of long fingers. The highest coordination is generally between middle and ring fingers. The TPS value between thumb and long finger tendons (around 20–30% on average) is lower than that observed among long fingers. Generally, the coordination between the thumb and little finger flexion is lower than that between the thumb and index finger flexion. In the case of the IMMA hand, the TPS values for the pair of tendons of the thumb (mean value, 47%) are on average higher than those for the pairs between thumb and long fingers tendons but lower than the values for the tendon pairs of long fingers.

**Figure 5:**
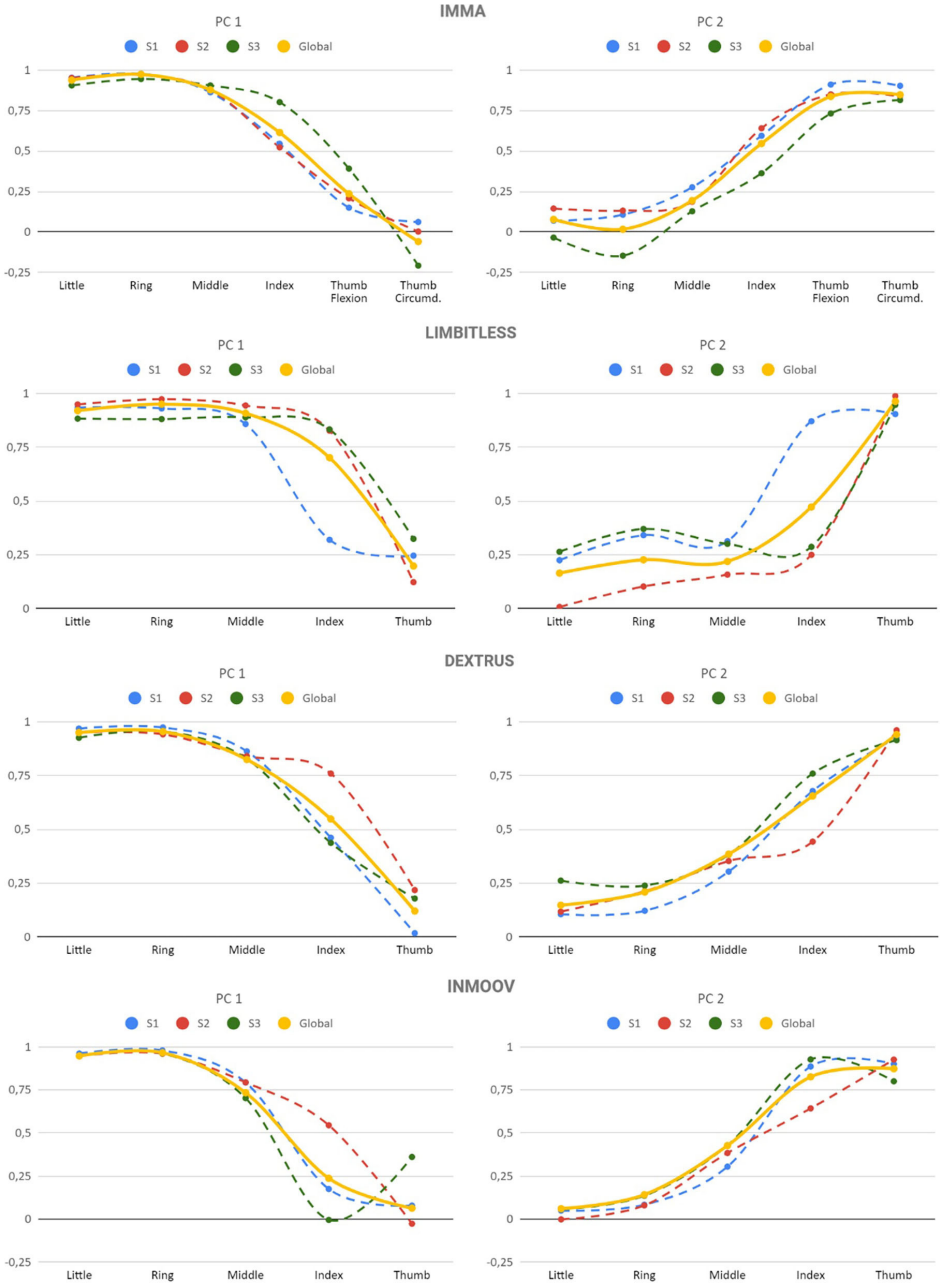
Eigenvectors (spline connected) for the Principal Components (PCs) of the data matrix of tendon displacements for the hands analyzed. The rotated component matrix was obtained from a PCA as the extraction method and using the Varimax with Kaiser normalization as the rotation method.

The results of the PCA show that, for all the hands, two PCs explained close to or more than 80% of the variance. Table 7 shows the cumulative variance explained with one or two PCs for the PCAs on the tendon displacement data matrices, both including the data for all the subjects together (Global) and for each subject independently (S1, S2, and S3). Figure 5 shows the principal components (PCs) obtained. For the IMMA hand, a very similar pattern is obtained for all the subjects in coherence with the result that the subject effect on TPSs is non-significant for this hand. However, in the case of the other hands, as the subject was significant in TPSs, the PCs have small variations, especially for the index finger, depending on the subject. Generally, the first PC explains the movement of the four long fingers, where little and ring fingers have almost the same score, and for the middle finger, it is a little lower. The movement of the index finger is split between the first and second PCs, in different proportions depending on the hand, and the second PC represents the movement of the thumb and the remaining part of the index.

**Table 7:** Cumulative variance explained by the PCs obtained in the PCAs.

**Hand**	**PC1 (%)**				**PC2 (%)**			
	**S1**	**S2**	**S3**	**Global**	**S1**	**S2**	**S3**	**Global**
IMMA	48.8	48.8	56.1	50.5	83.6	80.5	78.9	79.8
Limbitless	52.6	68.6	62.8	62.0	89.4	90.0	88.2	87.5
Dextrus	56.7	62.4	53.5	56.1	85.5	88.4	87.1	86.6
InMoov	50.9	54.9	48.9	48.6	84.9	83.4	83.0	81.6

**Figure 6:**
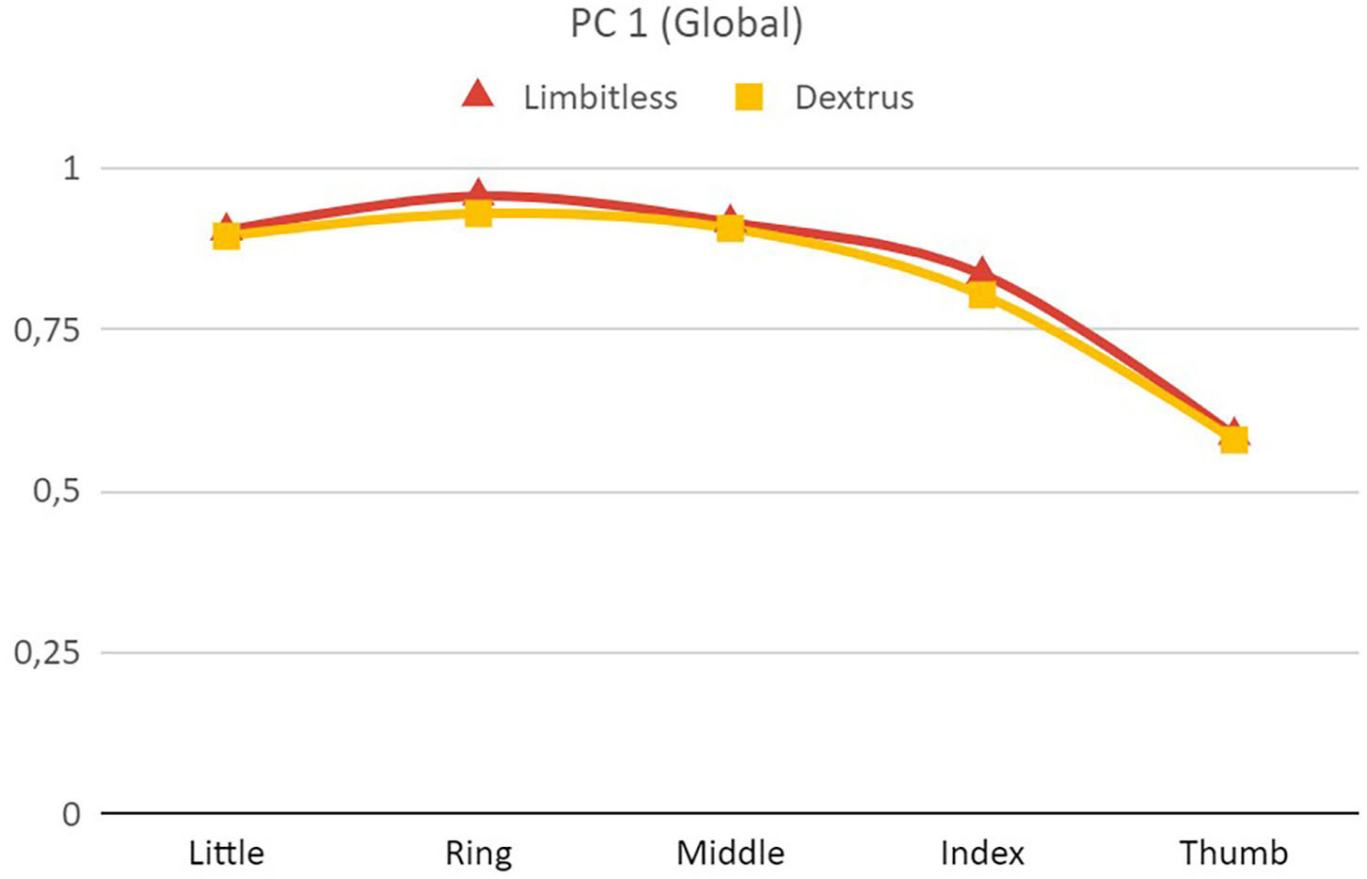
Eigenvectors (spline connected) for the Principal Component (PC) of the Limbitless and Dextrus v2.0 hands, where the component matrix was obtained from a PCA as the extraction method based on eigenvalue >1.

An additional PCA with extraction method based on eigenvalue >1 and including the data of all the subjects (Global) resulted in just one PC only for the Limbitless and Dextrus v2.0. This PC explained 72.1% of the variance for Limbitless and 69.3% for Dextrus v2.0. Figure 6 shows the similar pattern obtained for these two cases. Therefore, a common strategy for the transmission, actuation, and control systems might be used in these hands.

## Discussion

In this study, the grasping ability of four TDPHs has been analyzed and compared using an ABA. All the tendons of the artificial hands were actuated independently by healthy human subjects, that is, excluding from the comparison the specific control or actuation implementation with potential users with an amputation. For this reason, it should be noted that a worse performance can be expected with these final users because of the less efficient feedback, control strategy, and actuation system. As stated earlier, the objective of using this strategy was to compare the mechanical design of the hands taking advantage of the human brain control while obtaining the data directly from the artificial hand. Precluding any control setback not only encouraged us to use an ABA naturally moved by fingers but also to avoid any extrapolated learning from one trial to another in the design of the experiments within the same three subjects. The interval between the tests of two different artificial hands by the same subject was long enough (80 days on average), and, in the meantime, the subjects did not use the ABA to thus avoid learning effects between the tests of two different hands. Moreover, the absence of this effect can be evinced by the fact that the IMMA hand, which was the hand tested in the first instance, obtained the best GAS. All in all, the differences observed in grasping ability with respect to the human hand are attributed to several factors such as a lower friction coefficient on the hand contact surfaces, the lack of dexterity because of the underactuation, or the difficulty in accomplishing some hand postures, including the limitations for achieving a correct thumb opposition.

The fact that the IMMA hand scored the highest GAS is attributed mainly to two factors: it has an additional DoA for thumb circumduction, and it uses selected materials for the different parts of the hand. This result seems reasonable because the IMMA hand was partially inspired by other versions of the Limbitless and Dextrus hands, in an attempt to discern in a well-reasoned way the most advantageous design details of each one, as if it were some evolutionary process. The lowest GAS of the Dextrus v2.0 hand may be attributed to the use of a flexible material to generate a unibody rubberized hand, which has two main consequences: the percentage of infill used affects the maximum force exerted before the hand becomes warped, and the behavior of the joints in each digit is established early on in the printing process. For example, the excessive ease with which the DIP joint can be bent on the printed model made some precision grasps cumbersome. In the other models, the replacement and customization of a joint with more infill is straightforward.

With regard to the mechanical design of the hands, several improvements should be implemented in order to increase their functionality to be able to perform the most common GTs in ADLs. They should be especially focused on improving the grasping stability because, as shown in the results, holding the object securely under motion of the hands was the most challenging matter. In the case of the extension grip, it would be useful to have extensor tendons acting in a complementary way or to design joints with different bending stiffnesses, higher for DIP than for MCP joints. These mechanical solutions may make it possible to keep the distal segments of the fingers extended while flexing their MCP joints. In the case of pulp pinch, lateral pinch, diagonal volar grip, and tripod pinch, the orientation of the thumb as regards its opposition to the long fingers should be optimized. For this purpose, the authors (Pérez-González and Llop-Harillo, 2020) developed a computational method to optimize the thumb's kinematic chain (base placement, link lengths, and joint orientation angles) of an artificial hand based on its performance in the Kapandji opposition test (Kapandji, 1986) used in functional evaluations of the human hand. For the spherical, cylindrical, and diagonal volar grips, in addition to the thumb opposition, the palm opposition is also very relevant. Instead of a flat palm design, a more human-shaped one, achieved by mimicking both thenar and hypothenar eminences, should improve the performance for these GTs. The reachable grasping force is limiting for power grasps such as diagonal volar, extension, spherical, and cylindrical grips, especially when grasping big and heavy objects. In TDPHs, the mechanical advantage diminishes as the hand closes, meaning that a more limited grasping force can be exerted with the same pulling force at the tendons (Andrés et al., 2019). Decreasing the friction of the tendons along their path may improve the mechanical advantage (namely, the output to input force ratio) and thus this grasping force. Moreover, the selection of materials with good compliance and friction coefficient for the hand–objects contact areas is a key point to improve the stability of these grasps.

Regardless of the performance of the four TDPHs analyzed, the fact that the effect of the subject was non-significant on both GAS and partial GAS evinces that a single subject can be used to evaluate the grasping ability of artificial hands using this ABA and the AHAP. This observation may be useful when it comes to evaluating a mechanical design itself, to make the redesign and reevaluation process faster. A power analysis on GAS with a sample size of three subjects, minimum power of 0.8, significance level alpha of 0.05, and standard deviation of 2.46% (the mean value of the standard deviation among subjects for the tested hands), indicated a minimum detectable difference on GAS of 3.97%. This value seems reasonable in clinical terms because differences in GAS below this value will probably not have a significant impact on the hand performance. A reasonable power in the test with a limited number of subjects is possible due to the within-subjects full factorial design undertaken.

However, related to brain control, the motion synergies between fingers of the four TDPHs have also been analyzed and compared, and they yielded different outcomes. The ANOVA tests on TPS showed that the effect of the subject was non-significant for the IMMA hand, but it was significant for the other hands. This means that, for the case of the IMMA hand, the analyses of TPSs and PCA could be performed globally with all the data or according to one of the subjects. However, for the other hands, as the subject was found to be significant in TPS, differences appear, mainly in the coordination between the tendons of the thumb and long fingers and in the scores of the PCs for the index finger. This may be related to these hands having one less DoA, which makes it more cumbersome for them to achieve certain GTs and therefore forcing the subjects to find tricky ways to solve problems. That said, in a further step of having to design the control system of a hand model, it is of interest to consider the model having the best AHAP score for each task. For example, the training of a neural network with this previous selection of models for each task would make the process faster, yet this is far beyond the scope of this paper.

It is relevant to highlight the fact that the results obtained in this study on TPSs are in accordance with other studies in the literature performed with the human hand. Namely, Santello et al. (1998) obtained a high correlation between MCP angles of the long fingers in all subjects, the correlation being greater for adjacent fingers. The same was also obtained for their PIP angles. Moreover, the results of the PCA obtained in the present paper, in which the first PC stands for the movement of the four long fingers and the second one mainly for the movement of the thumb, are in agreement with a recent study by Gracia Ibañez (2016), where five PCs explained the synergies of the human hand performing ADLs. In that study, the first PC corresponds to the flexion of the interphalangeal joints of the fingers, and the last two PCs represent the movement of the thumb: the lateral opposition of the thumb to the index and the pad-to-pad opposition of the thumb to the little finger.

As can be seen from the information contained in Table 1 about the current design characteristics of the artificial hands, the Limbitless hand is actuated with only one motor. This actuation method is worthwhile, as one PC explains more than 70% of the variance observed for this hand. The other hands analyzed are actuated with five motors (Dextrus v2.0 and InMoov) or its actuation is not defined (IMMA). The PCs obtained in this study could be used to couple the actuation for some DoFs for these hands. This underactuation is essential to simplify the control of the hand, especially for affordable designs. The actuation coupling can be implemented at the software level, with an algorithm to reproduce the PCs and/or at the hardware level, through the design of the mechanical transmission. The dimensionality reduction using only one or two motors to actuate these hands should not affect their dexterity significantly. Indeed, some of the currently existing prostheses use only two motors, one to actuate the four long fingers and the other to actuate the thumb (Weiner et al., 2018). In other cases, three motors are used (Huang et al., 2006): one for the thumb; one for the middle, ring, and little finger; and one for the index finger. The actuation of middle, ring, and little fingers using the same motor is consistent with the results in Figure 5, as the scores corresponding to these fingers are quite similar. The use of an independent motor for the index makes sense because the score for this finger (Figure 5) has an intermediate value for the two PCs. The use of just one motor to operate all fingers takes dimensionality reduction to its maximum expression (Catalano et al., 2014). The methods employed in this study could help with this dimensionality reduction and thus with the mechanical design, testing, and control of the underactuation in TDPHs.

## Conclusions

In this study, an experimental comparison of the grasping ability of four different affordable anthropomorphic prosthetic hands has been undertaken using the AHAP benchmark. The grasping ability ranged between 48 and 57% with respect to that of the human hand, the best result being obtained by the IMMA hand. This is probably due to the additional DoA for circumduction of the thumb, not present in the other models, and also to the use of selected materials for the different parts of the hand. The hands exhibited better performance for non-grasping postures, such as index pointing or platform, and for hook and tripod pinch grasps. Worse performance was seen for extension grip, pulp pinch, spherical grip, and cylindrical grip. To improve the design of TDPHs, especially in terms of their grasping stability, which is the most challenging issue observed in this study, several aspects should be considered: (i) the orientation and position of the thumb for a correct thumb opposition; (ii) an appropriate mechanical solution for keeping the distal segments of the fingers extended while flexing their MCP joints; (iii) the maximization of the thumb mobility with additional DoAs while maintaining an easy actuation control; (iv) a human-shaped palm that provides palm opposition; (v) the grasping force exerted bearing in mind the mechanical advantage and the percentage of infill used, especially in flexible materials; and (vi) the compliance and friction coefficient of the materials used in the hand contact surfaces. The effect of the subject on the control of TDPHs when using an ABA has been analyzed. It has been shown that the effect of the subject on the GAS obtained was non-significant, revealing that a single subject could be used to evaluate hand prostheses using the ABA presented and the AHAP. However, the motion synergies were different for some of the users and the hands analyzed, especially in the coordination between the thumb and long fingers. The analysis of the synergies in the motion of the tendons used to actuate the hands showed that the actuation and control systems could be designed in order to couple some DoFs, due to the important correlations observed in adjacent long fingers. Two PCs are enough to explain more than 80% of the variability observed in the motions of the tendons for all the hands, with the first PC accounting for the movement of the four long fingers and the second PC explaining the movement of the thumb and the remaining part of the index finger. The scores of these PCs can be useful both for the design of transmission systems to underactuate the hand and for the design of the control system. Further research should address this underactuated design for the IMMA hand, designed by the authors, where the number of actuators could be limited to two motors while maintaining the dexterity of the hand to a significant extent.

## Data Availability Statement

All datasets generated for this study are included in the article/Supplementary Material.

## Ethics Statement

The studies involving human participants were reviewed and approved by Ethics Committee of the Universitat Jaume I (CD/006/2019). The patients/participants provided their written informed consent to participate in this study.

## Author Contributions

IL-H designed the study, primary writer of the manuscript, planned the experiments and analyzed, and interpreted the data. JA-E and IL-H selected and adapted the hands to analyze. AP-G and IL-H redesigned the able-bodied adaptor. AP-G supervised the entire research process. All authors conducted the experiments, read, revised, and approved the final manuscript.

## Conflict of Interest

The authors declare that the research was conducted in the absence of any commercial or financial relationships that could be construed as a potential conflict of interest.

## Supplementary Material

Table S1 Shows the scores for each step of each repetition of the different tasks of the AHAP obtained for each hand analyzed and each subject.

Supplementary Data Sheet 1 Displacement of the different tendons, for each hand analyzed and each subject, while performing the AHAP with the ABA. The data from each experiment are stored in a cell array divided into tasks (26 rows) and repetitions (3 columns). Inside each cell there is a matrix whose columns correspond to: time in seconds (first column), displacement in mm (columns 2–7) for the little, ring, middle and index fingers, thumb flexion and thumb abduction. The Matlab® files are named as POT_AAA_SX.mat, where AAA are the first three letters of the name of the hand and X is the number of the subject.

## Abbreviations

3D: three-dimensional
ABA: able-bodied adaptor
ABS: acrylonitrile butadiene styrene
ADL: activity of daily living
AHAP: Anthropomorphic Hand Assessment Protocol
ANOVA: analysis of variance
BBT: Box and Block Test
CAD: Computer-Aided Design
CC: Correlation Coefficient
CG: cylindrical grip
CMC: carpometacarpal
DIP: distal interphalangeal
DIY: do-it-yourself
DoA: degree of actuation
DoF: degree of freedom
DVG: diagonal volar grip
EG: extension grip
FDM: fused deposition modeling
GAS: Grasping Ability Score
GT: grasp type
H: hook
IP: index pointing/pressing
LP: lateral pinch
MCP: metacarpophalangeal
NHPT: Nine-Hole Peg Test
P: platform
PC: principal component
PCA: Principal Component Analysis
PIP: proximal interphalangeal
PLA: polylactic acid
PP: pulp pinch
SG: spherical grip
SHAP: Southampton Hand Assessment Procedure
TDPHs: tendon-driven prosthetic hands
TP: tripod pinch
TPS: tendon-pair synergy
TPU: thermoplastic polyurethane
UJI: Universitat Jaume I.
