# Supplementary material for: Grasping Ability and Motion Synergies in Affordable Tendon-Driven Prosthetic Hands Controlled by Able-Bodied Subjects
Source: Front Neurorobot. 2020 Aug 26;14:57. doi: 10.3389/fnbot.2020.00057 (PMC7480172; doi:10.3389/fnbot.2020.00057)
Supplement: Table S1 — Shows the scores for each step of each repetition of the different tasks of the AHAP obtained for each hand analyzed and each subject. [file Table_1.DOCX]

Supplementary Material

**Supplementary Table 1.** Scores for each step of each repetition of the different tasks of the AHAP obtained for each hand analyzed and each subject.

| Hand | Subject | T | Posture/Grasp type | Object | Grasping | | | Maintaining | | |
| --- | --- | --- | --- | --- | --- | --- | --- | --- | --- | --- |
|  |  |  |  |  | Trial 1 | Trial 2 | Trial 3 | Trial 1 | Trial 2 | Trial 3 |
| IMMA | 1 | 1 | Hook | Skillet lid | 1 | 1 | 1 | 0.5 | 0.5 | 0.5 |
|  |  | 2 | Spherical grip | Plastic apple | 1 | 0.5 | 1 | 1 | 0 | 0 |
|  |  | 3 | Tripod pinch | Large marker | 1 | 1 | 1 | 0 | 1 | 1 |
|  |  | 4 | Extension grip | Plate | 0.5 | 0.5 | 0.5 | 0 | 0 | 0 |
|  |  | 5 | Cylindrical grip | Chips can | 1 | 1 | 1 | 0 | 0 | 0 |
|  |  | 6 | Diagonal volar grip | Phillips screwdriver | 0.5 | 0.5 | 0.5 | 1 | 1 | 1 |
|  |  | 7 | Lateral pinch | Bowl | 1 | 1 | 1 | 0 | 0 | 0.5 |
|  |  | 8 | Pulp pinch | Small marker | 0.5 | 0.5 | 0.5 | 0 | 0 | 0 |
|  |  | 9 | Index pointing/pressing | Timer | 1 | 1 | 1 | 1 | 0 | 1 |
|  |  | 10 | Hook | Pitcher base | 1 | 1 | 1 | 0.5 | 0.5 | 0.5 |
|  |  | 11 | Spherical grip | Softball | 1 | 1 | 1 | 0 | 0 | 0 |
|  |  | 12 | Tripod pinch | Tuna can | 1 | 1 | 1 | 0 | 0 | 0 |
|  |  | 13 | Extension grip | Cracker box | 0 | 0 | 0 | 0 | 0 | 0 |
|  |  | 14 | Cylindrical grip | Coffee can | 1 | 1 | 1 | 0 | 0 | 0 |
|  |  | 15 | Diagonal volar grip | Spatula | 0.5 | 0.5 | 0.5 | 0.5 | 0.5 | 0.5 |
|  |  | 16 | Lateral pinch | XS Clamp | 1 | 1 | 0.5 | 1 | 0.5 | 0.5 |
|  |  | 17 | Pulp pinch | Plastic pear | 0 | 1 | 0 | 0 | 0 | 0 |
|  |  | 18 | Platform | Plate | 1 | 1 | 1 | - | - | - |
|  |  | 19 | Hook | Wood blocks with rope | 1 | 1 | 1 | 0.5 | 0.5 | 0.5 |
|  |  | 20 | Spherical grip | Mini soccer ball | 1 | 0 | 1 | 0 | 0 | 0 |
|  |  | 21 | Tripod pinch | Golf ball | 1 | 1 | 0.5 | 1 | 0.5 | 1 |
|  |  | 22 | Extension grip | Pudding box | 1 | 1 | 1 | 0 | 0 | 0 |
|  |  | 23 | Cylindrical grip | Power drill | 1 | 1 | 1 | 0.5 | 0 | 0 |
|  |  | 24 | Diagonal volar grip | Skillet | 0 | 0 | 0 | 0 | 0 | 0 |
|  |  | 25 | Lateral pinch | Key | 1 | 1 | 1 | 1 | 1 | 1 |
|  |  | 26 | Pulp pinch | Washer 10mm | 1 | 0.5 | 1 | 0 | 0.5 | 1 |
| IMMA | 2 | 1 | Hook | Skillet lid | 1 | 1 | 1 | 0.5 | 0.5 | 0.5 |
|  |  | 2 | Spherical grip | Plastic apple | 1 | 1 | 1 | 0 | 1 | 0.5 |
|  |  | 3 | Tripod pinch | Large marker | 1 | 1 | 1 | 1 | 0 | 1 |
|  |  | 4 | Extension grip | Plate | 1 | 0 | 1 | 0 | 0 | 0 |
|  |  | 5 | Cylindrical grip | Chips can | 1 | 1 | 0 | 0 | 1 | 0 |
|  |  | 6 | Diagonal volar grip | Phillips screwdriver | 0.5 | 0.5 | 0.5 | 1 | 1 | 0.5 |
|  |  | 7 | Lateral pinch | Bowl | 1 | 1 | 1 | 0 | 0.5 | 0.5 |
|  |  | 8 | Pulp pinch | Small marker | 0 | 0 | 0 | 0 | 0 | 0 |
|  |  | 9 | Index pointing/pressing | Timer | 1 | 1 | 0.5 | 1 | 1 | 1 |
|  |  | 10 | Hook | Pitcher base | 1 | 1 | 1 | 0.5 | 0.5 | 0.5 |
|  |  | 11 | Spherical grip | Softball | 1 | 1 | 1 | 0 | 0 | 0 |
|  |  | 12 | Tripod pinch | Tuna can | 1 | 1 | 1 | 0 | 0 | 0 |
|  |  | 13 | Extension grip | Cracker box | 0.5 | 0.5 | 0.5 | 0 | 0 | 0 |
|  |  | 14 | Cylindrical grip | Coffee can | 0.5 | 0.5 | 0.5 | 0 | 0 | 0 |
|  |  | 15 | Diagonal volar grip | Spatula | 0.5 | 0.5 | 0.5 | 1 | 1 | 1 |
|  |  | 16 | Lateral pinch | XS Clamp | 1 | 1 | 1 | 1 | 1 | 1 |
|  |  | 17 | Pulp pinch | Plastic pear | 0 | 0 | 1 | 0 | 0 | 0 |
|  |  | 18 | Platform | Plate | 1 | 1 | 1 | - | - | - |
|  |  | 19 | Hook | Wood blocks with rope | 1 | 1 | 1 | 0.5 | 0.5 | 0.5 |
|  |  | 20 | Spherical grip | Mini soccer ball | 1 | 0.5 | 1 | 0 | 0 | 0 |
|  |  | 21 | Tripod pinch | Golf ball | 1 | 1 | 1 | 1 | 1 | 1 |
|  |  | 22 | Extension grip | Pudding box | 1 | 1 | 1 | 0 | 0 | 0 |
|  |  | 23 | Cylindrical grip | Power drill | 1 | 1 | 1 | 0 | 0 | 0 |
|  |  | 24 | Diagonal volar grip | Skillet | 0 | 0 | 0 | 0 | 0 | 0 |
|  |  | 25 | Lateral pinch | Key | 1 | 1 | 1 | 0.5 | 0.5 | 1 |
|  |  | 26 | Pulp pinch | Washer 10mm | 1 | 0.5 | 1 | 1 | 1 | 1 |
| IMMA | 3 | 1 | Hook | Skillet lid | 1 | 1 | 1 | 0.5 | 0.5 | 0.5 |
|  |  | 2 | Spherical grip | Plastic apple | 1 | 1 | 1 | 0 | 0 | 0 |
|  |  | 3 | Tripod pinch | Large marker | 1 | 1 | 0 | 1 | 1 | 0 |
|  |  | 4 | Extension grip | Plate | 0.5 | 0.5 | 0.5 | 0 | 0 | 0 |
|  |  | 5 | Cylindrical grip | Chips can | 1 | 1 | 1 | 0 | 0 | 0 |
|  |  | 6 | Diagonal volar grip | Phillips screwdriver | 0.5 | 0.5 | 0.5 | 1 | 1 | 1 |
|  |  | 7 | Lateral pinch | Bowl | 1 | 1 | 1 | 0.5 | 0.5 | 0.5 |
|  |  | 8 | Pulp pinch | Small marker | 0 | 0 | 0 | 0 | 0 | 0 |
|  |  | 9 | Index pointing/pressing | Timer | 1 | 1 | 1 | 1 | 1 | 1 |
|  |  | 10 | Hook | Pitcher base | 1 | 1 | 1 | 1 | 0.5 | 0.5 |
|  |  | 11 | Spherical grip | Softball | 1 | 0.5 | 0.5 | 0 | 0 | 0 |
|  |  | 12 | Tripod pinch | Tuna can | 1 | 1 | 1 | 1 | 0 | 0 |
|  |  | 13 | Extension grip | Cracker box | 0 | 0 | 0 | 0 | 0 | 0 |
|  |  | 14 | Cylindrical grip | Coffee can | 1 | 1 | 1 | 0 | 0 | 0 |
|  |  | 15 | Diagonal volar grip | Spatula | 0.5 | 0.5 | 0.5 | 1 | 1 | 1 |
|  |  | 16 | Lateral pinch | XS Clamp | 1 | 1 | 1 | 1 | 1 | 1 |
|  |  | 17 | Pulp pinch | Plastic pear | 1 | 1 | 1 | 0.5 | 0 | 1 |
|  |  | 18 | Platform | Plate | 1 | 1 | 1 | - | - | - |
|  |  | 19 | Hook | Wood blocks with rope | 1 | 1 | 1 | 0.5 | 0.5 | 0.5 |
|  |  | 20 | Spherical grip | Mini soccer ball | 1 | 0.5 | 0.5 | 0 | 0 | 0 |
|  |  | 21 | Tripod pinch | Golf ball | 1 | 1 | 0.5 | 1 | 1 | 1 |
|  |  | 22 | Extension grip | Pudding box | 1 | 0.5 | 1 | 0 | 0 | 0 |
|  |  | 23 | Cylindrical grip | Power drill | 1 | 1 | 1 | 0 | 0 | 0 |
|  |  | 24 | Diagonal volar grip | Skillet | 0 | 0 | 0 | 0 | 0 | 0 |
|  |  | 25 | Lateral pinch | Key | 1 | 1 | 1 | 0.5 | 1 | 1 |
|  |  | 26 | Pulp pinch | Washer 10mm | 1 | 0 | 0.5 | 0 | 0 | 1 |
| Limbitless | 1 | 1 | Hook | Skillet lid | 1 | 1 | 1 | 0.5 | 0.5 | 0.5 |
|  |  | 2 | Spherical grip | Plastic apple | 0 | 1 | 1 | 0 | 0 | 0 |
|  |  | 3 | Tripod pinch | Large marker | 0 | 1 | 0 | 0 | 0 | 0 |
|  |  | 4 | Extension grip | Plate | 1 | 1 | 1 | 0 | 0 | 0 |
|  |  | 5 | Cylindrical grip | Chips can | 0.5 | 0.5 | 0.5 | 0 | 0 | 0 |
|  |  | 6 | Diagonal volar grip | Phillips screwdriver | 0.5 | 0.5 | 0.5 | 1 | 1 | 1 |
|  |  | 7 | Lateral pinch | Bowl | 1 | 1 | 1 | 0.5 | 0.5 | 0 |
|  |  | 8 | Pulp pinch | Small marker | 0 | 0 | 0 | 0 | 0 | 0 |
|  |  | 9 | Index pointing/pressing | Timer | 1 | 1 | 1 | 1 | 1 | 1 |
|  |  | 10 | Hook | Pitcher base | 1 | 1 | 1 | 0.5 | 0.5 | 0.5 |
|  |  | 11 | Spherical grip | Softball | 1 | 1 | 1 | 0 | 0 | 0 |
|  |  | 12 | Tripod pinch | Tuna can | 1 | 1 | 1 | 0 | 0.5 | 0 |
|  |  | 13 | Extension grip | Cracker box | 0 | 0 | 0 | 0 | 0 | 0 |
|  |  | 14 | Cylindrical grip | Coffee can | 0.5 | 0.5 | 0.5 | 0 | 0 | 0 |
|  |  | 15 | Diagonal volar grip | Spatula | 0.5 | 0.5 | 0.5 | 1 | 1 | 1 |
|  |  | 16 | Lateral pinch | XS Clamp | 0.5 | 0.5 | 0.5 | 1 | 1 | 1 |
|  |  | 17 | Pulp pinch | Plastic pear | 0 | 0.5 | 0 | 0 | 1 | 0 |
|  |  | 18 | Platform | Plate | 0 | 0 | 0 | - | - | - |
|  |  | 19 | Hook | Wood blocks with rope | 1 | 1 | 1 | 0.5 | 0.5 | 0.5 |
|  |  | 20 | Spherical grip | Mini soccer ball | 0 | 0 | 0 | 0 | 0 | 0 |
|  |  | 21 | Tripod pinch | Golf ball | 1 | 1 | 1 | 1 | 1 | 0 |
|  |  | 22 | Extension grip | Pudding box | 1 | 1 | 1 | 0 | 0 | 0 |
|  |  | 23 | Cylindrical grip | Power drill | 1 | 1 | 1 | 1 | 0.5 | 0.5 |
|  |  | 24 | Diagonal volar grip | Skillet | 0.5 | 0.5 | 0.5 | 0 | 0 | 0 |
|  |  | 25 | Lateral pinch | Key | 1 | 1 | 1 | 1 | 1 | 0.5 |
|  |  | 26 | Pulp pinch | Washer 10mm | 0 | 0 | 0 | 0 | 0 | 0 |
| Limbitless | 2 | 1 | Hook | Skillet lid | 1 | 1 | 1 | 0.5 | 0.5 | 1 |
|  |  | 2 | Spherical grip | Plastic apple | 0 | 0 | 0 | 0 | 0 | 0 |
|  |  | 3 | Tripod pinch | Large marker | 0 | 1 | 1 | 0 | 1 | 1 |
|  |  | 4 | Extension grip | Plate | 1 | 1 | 1 | 0 | 0 | 0 |
|  |  | 5 | Cylindrical grip | Chips can | 0.5 | 0.5 | 0.5 | 0 | 0 | 0 |
|  |  | 6 | Diagonal volar grip | Phillips screwdriver | 0.5 | 0.5 | 0.5 | 1 | 1 | 1 |
|  |  | 7 | Lateral pinch | Bowl | 1 | 1 | 1 | 0.5 | 0.5 | 1 |
|  |  | 8 | Pulp pinch | Small marker | 0 | 0 | 0 | 0 | 0 | 0 |
|  |  | 9 | Index pointing/pressing | Timer | 1 | 1 | 1 | 1 | 1 | 1 |
|  |  | 10 | Hook | Pitcher base | 1 | 1 | 1 | 1 | 0.5 | 0.5 |
|  |  | 11 | Spherical grip | Softball | 1 | 1 | 1 | 0 | 0 | 0 |
|  |  | 12 | Tripod pinch | Tuna can | 1 | 1 | 1 | 0 | 0 | 0 |
|  |  | 13 | Extension grip | Cracker box | 0 | 0 | 0 | 0 | 0 | 0 |
|  |  | 14 | Cylindrical grip | Coffee can | 0.5 | 0.5 | 0.5 | 0 | 0 | 0 |
|  |  | 15 | Diagonal volar grip | Spatula | 0.5 | 0.5 | 0.5 | 1 | 1 | 1 |
|  |  | 16 | Lateral pinch | XS Clamp | 0 | 0 | 0.5 | 0 | 0 | 1 |
|  |  | 17 | Pulp pinch | Plastic pear | 0.5 | 0.5 | 0.5 | 0.5 | 0 | 1 |
|  |  | 18 | Platform | Plate | 0 | 0 | 0 | - | - | - |
|  |  | 19 | Hook | Wood blocks with rope | 1 | 1 | 1 | 0.5 | 0.5 | 0.5 |
|  |  | 20 | Spherical grip | Mini soccer ball | 0 | 0 | 0 | 0 | 0 | 0 |
|  |  | 21 | Tripod pinch | Golf ball | 1 | 1 | 1 | 1 | 1 | 1 |
|  |  | 22 | Extension grip | Pudding box | 1 | 1 | 1 | 0 | 0.5 | 0 |
|  |  | 23 | Cylindrical grip | Power drill | 1 | 1 | 1 | 1 | 0.5 | 1 |
|  |  | 24 | Diagonal volar grip | Skillet | 0.5 | 0.5 | 0.5 | 0 | 0 | 0 |
|  |  | 25 | Lateral pinch | Key | 1 | 1 | 1 | 1 | 0.5 | 0.5 |
|  |  | 26 | Pulp pinch | Washer 10mm | 1 | 1 | 1 | 1 | 1 | 0.5 |
| Limbitless | 3 | 1 | Hook | Skillet lid | 1 | 1 | 1 | 0.5 | 0.5 | 0.5 |
|  |  | 2 | Spherical grip | Plastic apple | 0 | 1 | 1 | 0 | 0 | 0 |
|  |  | 3 | Tripod pinch | Large marker | 1 | 0 | 1 | 1 | 0 | 1 |
|  |  | 4 | Extension grip | Plate | 1 | 1 | 1 | 0 | 0 | 0 |
|  |  | 5 | Cylindrical grip | Chips can | 0.5 | 0.5 | 0.5 | 0 | 0 | 0 |
|  |  | 6 | Diagonal volar grip | Phillips screwdriver | 0.5 | 0.5 | 0.5 | 1 | 1 | 1 |
|  |  | 7 | Lateral pinch | Bowl | 1 | 1 | 1 | 0 | 0 | 0 |
|  |  | 8 | Pulp pinch | Small marker | 0 | 0 | 0 | 0 | 0 | 0 |
|  |  | 9 | Index pointing/pressing | Timer | 1 | 1 | 1 | 1 | 1 | 1 |
|  |  | 10 | Hook | Pitcher base | 1 | 1 | 1 | 1 | 0.5 | 1 |
|  |  | 11 | Spherical grip | Softball | 1 | 1 | 1 | 0 | 0 | 0 |
|  |  | 12 | Tripod pinch | Tuna can | 1 | 1 | 1 | 0 | 0 | 0.5 |
|  |  | 13 | Extension grip | Cracker box | 0 | 0 | 0 | 0 | 0 | 0 |
|  |  | 14 | Cylindrical grip | Coffee can | 0.5 | 0.5 | 0.5 | 0 | 0 | 0 |
|  |  | 15 | Diagonal volar grip | Spatula | 0.5 | 0.5 | 0.5 | 1 | 1 | 1 |
|  |  | 16 | Lateral pinch | XS Clamp | 0 | 0 | 0 | 0 | 0 | 0 |
|  |  | 17 | Pulp pinch | Plastic pear | 0.5 | 0.5 | 0.5 | 1 | 0 | 0 |
|  |  | 18 | Platform | Plate | 0 | 0 | 0 | - | - | - |
|  |  | 19 | Hook | Wood blocks with rope | 1 | 1 | 1 | 0.5 | 0.5 | 1 |
|  |  | 20 | Spherical grip | Mini soccer ball | 0 | 0 | 0 | 0 | 0 | 0 |
|  |  | 21 | Tripod pinch | Golf ball | 1 | 1 | 1 | 1 | 1 | 1 |
|  |  | 22 | Extension grip | Pudding box | 1 | 1 | 1 | 0 | 0 | 0 |
|  |  | 23 | Cylindrical grip | Power drill | 1 | 1 | 1 | 0.5 | 0.5 | 1 |
|  |  | 24 | Diagonal volar grip | Skillet | 0 | 0.5 | 0 | 0 | 0 | 0 |
|  |  | 25 | Lateral pinch | Key | 1 | 1 | 1 | 1 | 1 | 1 |
|  |  | 26 | Pulp pinch | Washer 10mm | 0 | 0 | 0 | 0 | 0 | 0 |
| Dextrus v2.0 | 1 | 1 | Hook | Skillet lid | 1 | 1 | 1 | 0.5 | 0.5 | 0.5 |
|  |  | 2 | Spherical grip | Plastic apple | 0.5 | 0.5 | 1 | 0 | 1 | 0 |
|  |  | 3 | Tripod pinch | Large marker | 1 | 1 | 1 | 1 | 0 | 1 |
|  |  | 4 | Extension grip | Plate | 0 | 0.5 | 0 | 0 | 0 | 0 |
|  |  | 5 | Cylindrical grip | Chips can | 1 | 1 | 1 | 0 | 0 | 0 |
|  |  | 6 | Diagonal volar grip | Phillips screwdriver | 0.5 | 0.5 | 0.5 | 1 | 1 | 1 |
|  |  | 7 | Lateral pinch | Bowl | 0.5 | 1 | 0.5 | 0 | 0 | 0 |
|  |  | 8 | Pulp pinch | Small marker | 0 | 0 | 0.5 | 0 | 0 | 0.5 |
|  |  | 9 | Index pointing/pressing | Timer | 1 | 1 | 1 | 1 | 1 | 1 |
|  |  | 10 | Hook | Pitcher base | 1 | 1 | 1 | 0.5 | 1 | 1 |
|  |  | 11 | Spherical grip | Softball | 0.5 | 0.5 | 1 | 0 | 0 | 0 |
|  |  | 12 | Tripod pinch | Tuna can | 1 | 0 | 0.5 | 0 | 0 | 0 |
|  |  | 13 | Extension grip | Cracker box | 0 | 0 | 0 | 0 | 0 | 0 |
|  |  | 14 | Cylindrical grip | Coffee can | 0.5 | 0.5 | 0.5 | 0 | 0 | 0 |
|  |  | 15 | Diagonal volar grip | Spatula | 0.5 | 0.5 | 0.5 | 1 | 1 | 0.5 |
|  |  | 16 | Lateral pinch | XS Clamp | 0.5 | 0.5 | 0.5 | 0.5 | 0.5 | 0.5 |
|  |  | 17 | Pulp pinch | Plastic pear | 0 | 0 | 0.5 | 0 | 0 | 0 |
|  |  | 18 | Platform | Plate | 1 | 1 | 1 | - | - | - |
|  |  | 19 | Hook | Wood blocks with rope | 1 | 1 | 1 | 0.5 | 0.5 | 1 |
|  |  | 20 | Spherical grip | Mini soccer ball | 0.5 | 0.5 | 0.5 | 0 | 0 | 0 |
|  |  | 21 | Tripod pinch | Golf ball | 1 | 1 | 1 | 0.5 | 1 | 1 |
|  |  | 22 | Extension grip | Pudding box | 0.5 | 0.5 | 0 | 0 | 0 | 0 |
|  |  | 23 | Cylindrical grip | Power drill | 1 | 0.5 | 0.5 | 0 | 0 | 0 |
|  |  | 24 | Diagonal volar grip | Skillet | 0 | 0 | 0 | 0 | 0 | 0 |
|  |  | 25 | Lateral pinch | Key | 0 | 0 | 0.5 | 0 | 0 | 0 |
|  |  | 26 | Pulp pinch | Washer 10mm | 0.5 | 0.5 | 0.5 | 0 | 0 | 0 |
| Dextrus v2.0 | 2 | 1 | Hook | Skillet lid | 1 | 1 | 1 | 0.5 | 0.5 | 0.5 |
|  |  | 2 | Spherical grip | Plastic apple | 1 | 1 | 1 | 0 | 0 | 0 |
|  |  | 3 | Tripod pinch | Large marker | 1 | 0 | 0.5 | 1 | 0 | 1 |
|  |  | 4 | Extension grip | Plate | 0.5 | 1 | 0.5 | 0 | 0 | 0 |
|  |  | 5 | Cylindrical grip | Chips can | 1 | 1 | 1 | 0 | 0 | 0 |
|  |  | 6 | Diagonal volar grip | Phillips screwdriver | 0.5 | 0.5 | 0.5 | 1 | 1 | 1 |
|  |  | 7 | Lateral pinch | Bowl | 1 | 1 | 1 | 0 | 0 | 0 |
|  |  | 8 | Pulp pinch | Small marker | 0 | 0 | 0 | 0 | 0 | 0 |
|  |  | 9 | Index pointing/pressing | Timer | 0 | 0 | 1 | 0 | 0 | 0 |
|  |  | 10 | Hook | Pitcher base | 1 | 1 | 1 | 0.5 | 0.5 | 0.5 |
|  |  | 11 | Spherical grip | Softball | 0.5 | 0 | 0.5 | 0 | 0 | 0 |
|  |  | 12 | Tripod pinch | Tuna can | 1 | 1 | 1 | 0 | 0 | 0 |
|  |  | 13 | Extension grip | Cracker box | 0 | 0 | 0 | 0 | 0 | 0 |
|  |  | 14 | Cylindrical grip | Coffee can | 1 | 1 | 1 | 0 | 0 | 0 |
|  |  | 15 | Diagonal volar grip | Spatula | 0.5 | 0.5 | 0.5 | 1 | 1 | 1 |
|  |  | 16 | Lateral pinch | XS Clamp | 0.5 | 0.5 | 0.5 | 0 | 1 | 1 |
|  |  | 17 | Pulp pinch | Plastic pear | 0.5 | 0.5 | 0.5 | 0 | 0 | 0 |
|  |  | 18 | Platform | Plate | 1 | 1 | 1 | - | - | - |
|  |  | 19 | Hook | Wood blocks with rope | 1 | 1 | 1 | 0.5 | 0.5 | 0.5 |
|  |  | 20 | Spherical grip | Mini soccer ball | 0 | 0.5 | 0 | 0 | 0 | 0 |
|  |  | 21 | Tripod pinch | Golf ball | 1 | 1 | 1 | 1 | 1 | 1 |
|  |  | 22 | Extension grip | Pudding box | 0.5 | 0.5 | 0.5 | 1 | 1 | 1 |
|  |  | 23 | Cylindrical grip | Power drill | 0.5 | 0.5 | 0.5 | 0 | 0 | 0 |
|  |  | 24 | Diagonal volar grip | Skillet | 0 | 0 | 0 | 0 | 0 | 0 |
|  |  | 25 | Lateral pinch | Key | 0 | 0.5 | 1 | 0 | 0.5 | 0 |
|  |  | 26 | Pulp pinch | Washer 10mm | 0.5 | 0.5 | 1 | 1 | 1 | 1 |
| Dextrus v2.0 | 3 | 1 | Hook | Skillet lid | 1 | 1 | 1 | 0.5 | 0.5 | 0.5 |
|  |  | 2 | Spherical grip | Plastic apple | 0.5 | 0.5 | 0.5 | 1 | 0 | 1 |
|  |  | 3 | Tripod pinch | Large marker | 0 | 1 | 1 | 0 | 1 | 1 |
|  |  | 4 | Extension grip | Plate | 0.5 | 0.5 | 0.5 | 0 | 0 | 0 |
|  |  | 5 | Cylindrical grip | Chips can | 1 | 1 | 1 | 0 | 0 | 0 |
|  |  | 6 | Diagonal volar grip | Phillips screwdriver | 0.5 | 0.5 | 0.5 | 1 | 1 | 1 |
|  |  | 7 | Lateral pinch | Bowl | 1 | 1 | 1 | 0 | 0 | 0 |
|  |  | 8 | Pulp pinch | Small marker | 0 | 0.5 | 0 | 0 | 1 | 0 |
|  |  | 9 | Index pointing/pressing | Timer | 1 | 1 | 1 | 1 | 1 | 1 |
|  |  | 10 | Hook | Pitcher base | 1 | 1 | 1 | 1 | 0.5 | 0.5 |
|  |  | 11 | Spherical grip | Softball | 1 | 0.5 | 1 | 1 | 0 | 1 |
|  |  | 12 | Tripod pinch | Tuna can | 1 | 1 | 1 | 0 | 0 | 0 |
|  |  | 13 | Extension grip | Cracker box | 0 | 0 | 0 | 0 | 0 | 0 |
|  |  | 14 | Cylindrical grip | Coffee can | 0.5 | 0.5 | 0.5 | 0 | 0 | 0 |
|  |  | 15 | Diagonal volar grip | Spatula | 0.5 | 0.5 | 0.5 | 1 | 1 | 1 |
|  |  | 16 | Lateral pinch | XS Clamp | 0 | 0.5 | 0.5 | 0 | 1 | 0.5 |
|  |  | 17 | Pulp pinch | Plastic pear | 0 | 0.5 | 0.5 | 0 | 1 | 1 |
|  |  | 18 | Platform | Plate | 1 | 1 | 1 | - | - | - |
|  |  | 19 | Hook | Wood blocks with rope | 1 | 1 | 1 | 0.5 | 0.5 | 0.5 |
|  |  | 20 | Spherical grip | Mini soccer ball | 0.5 | 0.5 | 0.5 | 0 | 0 | 0 |
|  |  | 21 | Tripod pinch | Golf ball | 1 | 1 | 1 | 1 | 0.5 | 1 |
|  |  | 22 | Extension grip | Pudding box | 0.5 | 0.5 | 0.5 | 0 | 0 | 0 |
|  |  | 23 | Cylindrical grip | Power drill | 0.5 | 0.5 | 0.5 | 0 | 0 | 0 |
|  |  | 24 | Diagonal volar grip | Skillet | 0 | 0 | 0 | 0 | 0 | 0 |
|  |  | 25 | Lateral pinch | Key | 1 | 1 | 0 | 0 | 0 | 0 |
|  |  | 26 | Pulp pinch | Washer 10mm | 0.5 | 0.5 | 0.5 | 1 | 1 | 1 |
| InMoov | 1 | 1 | Hook | Skillet lid | 1 | 1 | 1 | 0.5 | 0.5 | 0.5 |
|  |  | 2 | Spherical grip | Plastic apple | 0.5 | 0.5 | 0.5 | 0.5 | 1 | 1 |
|  |  | 3 | Tripod pinch | Large marker | 1 | 1 | 1 | 1 | 1 | 1 |
|  |  | 4 | Extension grip | Plate | 0 | 0 | 0 | 0 | 0 | 0 |
|  |  | 5 | Cylindrical grip | Chips can | 0.5 | 0.5 | 0.5 | 0 | 0 | 0 |
|  |  | 6 | Diagonal volar grip | Phillips screwdriver | 0.5 | 0.5 | 0.5 | 1 | 1 | 1 |
|  |  | 7 | Lateral pinch | Bowl | 1 | 1 | 1 | 0 | 0 | 0 |
|  |  | 8 | Pulp pinch | Small marker | 0 | 0 | 0 | 0 | 0 | 0 |
|  |  | 9 | Index pointing/pressing | Timer | 1 | 1 | 1 | 1 | 1 | 1 |
|  |  | 10 | Hook | Pitcher base | 1 | 1 | 1 | 1 | 0.5 | 1 |
|  |  | 11 | Spherical grip | Softball | 0.5 | 0.5 | 0.5 | 0 | 0 | 0 |
|  |  | 12 | Tripod pinch | Tuna can | 1 | 1 | 1 | 0 | 0 | 0 |
|  |  | 13 | Extension grip | Cracker box | 0.5 | 0 | 0 | 0 | 0 | 0 |
|  |  | 14 | Cylindrical grip | Coffee can | 0.5 | 0.5 | 0.5 | 0 | 0 | 0 |
|  |  | 15 | Diagonal volar grip | Spatula | 0.5 | 0.5 | 0.5 | 1 | 1 | 1 |
|  |  | 16 | Lateral pinch | XS Clamp | 1 | 1 | 0 | 1 | 1 | 0 |
|  |  | 17 | Pulp pinch | Plastic pear | 0 | 0.5 | 0 | 0 | 0 | 0 |
|  |  | 18 | Platform | Plate | 1 | 1 | 1 | - | - | - |
|  |  | 19 | Hook | Wood blocks with rope | 1 | 1 | 1 | 0.5 | 0.5 | 0.5 |
|  |  | 20 | Spherical grip | Mini soccer ball | 0.5 | 0.5 | 0.5 | 0 | 0 | 0 |
|  |  | 21 | Tripod pinch | Golf ball | 0.5 | 0.5 | 0.5 | 1 | 1 | 1 |
|  |  | 22 | Extension grip | Pudding box | 0.5 | 0.5 | 0.5 | 0 | 0 | 0 |
|  |  | 23 | Cylindrical grip | Power drill | 0.5 | 0.5 | 0.5 | 0 | 0 | 0 |
|  |  | 24 | Diagonal volar grip | Skillet | 0 | 0 | 0 | 0 | 0 | 0 |
|  |  | 25 | Lateral pinch | Key | 0.5 | 0.5 | 0.5 | 1 | 0.5 | 1 |
|  |  | 26 | Pulp pinch | Washer 10mm | 0.5 | 0 | 0.5 | 1 | 0 | 1 |
| InMoov | 2 | 1 | Hook | Skillet lid | 1 | 1 | 1 | 0.5 | 0.5 | 0.5 |
|  |  | 2 | Spherical grip | Plastic apple | 0.5 | 0.5 | 0.5 | 1 | 1 | 1 |
|  |  | 3 | Tripod pinch | Large marker | 0.5 | 0.5 | 0.5 | 1 | 1 | 1 |
|  |  | 4 | Extension grip | Plate | 0.5 | 0 | 0 | 0 | 0 | 0 |
|  |  | 5 | Cylindrical grip | Chips can | 0.5 | 0 | 0.5 | 0 | 0 | 0 |
|  |  | 6 | Diagonal volar grip | Phillips screwdriver | 0.5 | 0.5 | 0.5 | 1 | 1 | 1 |
|  |  | 7 | Lateral pinch | Bowl | 1 | 1 | 1 | 0 | 0 | 0 |
|  |  | 8 | Pulp pinch | Small marker | 0 | 0 | 0 | 0 | 0 | 0 |
|  |  | 9 | Index pointing/pressing | Timer | 1 | 1 | 1 | 1 | 1 | 1 |
|  |  | 10 | Hook | Pitcher base | 1 | 1 | 1 | 0.5 | 1 | 1 |
|  |  | 11 | Spherical grip | Softball | 0.5 | 0.5 | 0.5 | 0 | 0 | 0 |
|  |  | 12 | Tripod pinch | Tuna can | 1 | 1 | 1 | 0.5 | 0.5 | 0.5 |
|  |  | 13 | Extension grip | Cracker box | 0.5 | 0.5 | 0 | 0 | 0 | 0 |
|  |  | 14 | Cylindrical grip | Coffee can | 0 | 0.5 | 0 | 0 | 0 | 0 |
|  |  | 15 | Diagonal volar grip | Spatula | 0.5 | 0.5 | 0.5 | 1 | 1 | 1 |
|  |  | 16 | Lateral pinch | XS Clamp | 0.5 | 1 | 0 | 1 | 0 | 0 |
|  |  | 17 | Pulp pinch | Plastic pear | 0 | 0 | 0 | 0 | 0 | 0 |
|  |  | 18 | Platform | Plate | 1 | 1 | 1 | - | - | - |
|  |  | 19 | Hook | Wood blocks with rope | 1 | 1 | 1 | 0.5 | 0.5 | 0.5 |
|  |  | 20 | Spherical grip | Mini soccer ball | 0.5 | 0.5 | 0.5 | 0 | 0 | 0 |
|  |  | 21 | Tripod pinch | Golf ball | 1 | 1 | 0 | 1 | 1 | 0 |
|  |  | 22 | Extension grip | Pudding box | 0.5 | 0.5 | 0.5 | 0 | 0 | 0 |
|  |  | 23 | Cylindrical grip | Power drill | 0.5 | 0.5 | 0.5 | 0 | 0 | 0 |
|  |  | 24 | Diagonal volar grip | Skillet | 0 | 0 | 0 | 0 | 0 | 0 |
|  |  | 25 | Lateral pinch | Key | 1 | 1 | 1 | 1 | 1 | 1 |
|  |  | 26 | Pulp pinch | Washer 10mm | 0.5 | 0.5 | 0 | 1 | 0 | 0 |
| InMoov | 3 | 1 | Hook | Skillet lid | 1 | 1 | 1 | 0.5 | 0.5 | 0.5 |
|  |  | 2 | Spherical grip | Plastic apple | 0.5 | 0.5 | 0.5 | 1 | 1 | 1 |
|  |  | 3 | Tripod pinch | Large marker | 0.5 | 0.5 | 0.5 | 1 | 0.5 | 1 |
|  |  | 4 | Extension grip | Plate | 0.5 | 0.5 | 0.5 | 0 | 0 | 0 |
|  |  | 5 | Cylindrical grip | Chips can | 0.5 | 0.5 | 0.5 | 0 | 0 | 0 |
|  |  | 6 | Diagonal volar grip | Phillips screwdriver | 0.5 | 0.5 | 0.5 | 1 | 1 | 1 |
|  |  | 7 | Lateral pinch | Bowl | 1 | 1 | 1 | 0 | 0 | 0 |
|  |  | 8 | Pulp pinch | Small marker | 0 | 0 | 0 | 0 | 0 | 0 |
|  |  | 9 | Index pointing/pressing | Timer | 1 | 1 | 1 | 1 | 1 | 1 |
|  |  | 10 | Hook | Pitcher base | 1 | 1 | 1 | 1 | 0.5 | 1 |
|  |  | 11 | Spherical grip | Softball | 0.5 | 0.5 | 0.5 | 0 | 0 | 0 |
|  |  | 12 | Tripod pinch | Tuna can | 1 | 1 | 1 | 0 | 0 | 0 |
|  |  | 13 | Extension grip | Cracker box | 0.5 | 0.5 | 0.5 | 0 | 0 | 0 |
|  |  | 14 | Cylindrical grip | Coffee can | 0.5 | 0.5 | 0.5 | 0 | 0 | 0 |
|  |  | 15 | Diagonal volar grip | Spatula | 0.5 | 0.5 | 0.5 | 1 | 1 | 1 |
|  |  | 16 | Lateral pinch | XS Clamp | 0.5 | 0.5 | 0.5 | 1 | 1 | 1 |
|  |  | 17 | Pulp pinch | Plastic pear | 0 | 0 | 0 | 0 | 0 | 0 |
|  |  | 18 | Platform | Plate | 1 | 1 | 1 | - | - | - |
|  |  | 19 | Hook | Wood blocks with rope | 1 | 1 | 1 | 0.5 | 0.5 | 0.5 |
|  |  | 20 | Spherical grip | Mini soccer ball | 0.5 | 0.5 | 0.5 | 0 | 0 | 0 |
|  |  | 21 | Tripod pinch | Golf ball | 0.5 | 0.5 | 0.5 | 1 | 1 | 1 |
|  |  | 22 | Extension grip | Pudding box | 0.5 | 0.5 | 0.5 | 0 | 0 | 0 |
|  |  | 23 | Cylindrical grip | Power drill | 0.5 | 0.5 | 0.5 | 0 | 0 | 0 |
|  |  | 24 | Diagonal volar grip | Skillet | 0 | 0 | 0 | 0 | 0 | 0 |
|  |  | 25 | Lateral pinch | Key | 0.5 | 0.5 | 0.5 | 1 | 1 | 0.5 |
|  |  | 26 | Pulp pinch | Washer 10mm | 0.5 | 0.5 | 0.5 | 1 | 1 | 1 |
